# Multimodal spatiotemporal phenotyping of human retinal organoid development

**DOI:** 10.1038/s41587-023-01747-2

**Published:** 2023-05-08

**Authors:** Philipp Wahle, Giovanna Brancati, Christoph Harmel, Zhisong He, Gabriele Gut, Jacobo Sarabia del Castillo, Aline Xavier da Silveira dos Santos, Qianhui Yu, Pascal Noser, Jonas Simon Fleck, Bruno Gjeta, Dinko Pavlinić, Simone Picelli, Max Hess, Gregor W. Schmidt, Tom T. A. Lummen, Yanyan Hou, Patricia Galliker, David Goldblum, Marton Balogh, Cameron S. Cowan, Hendrik P. N. Scholl, Botond Roska, Magdalena Renner, Lucas Pelkmans, Barbara Treutlein, J. Gray Camp

**Affiliations:** 1 Department of Biosystems Science and Engineering, ETH Zürich, Basel, Switzerland; 2 Institute of Molecular and Clinical Ophthalmology Basel, Basel, Switzerland; 3 Department of Ophthalmology, University of Basel, Basel, Switzerland; 4 Department of Molecular Life Sciences, University of Zurich, Zurich, Switzerland; 5 Present Address: Institute of Human Biology (IHB), Roche Pharma Research and Early Development, Roche Innovation Center Basel, Basel, Switzerland

**Keywords:** Developmental neurogenesis, Transcriptomics, Stem-cell biotechnology, Multicellular systems

## Abstract

Organoids generated from human pluripotent stem cells provide experimental systems to study development and disease, but quantitative measurements across different spatial scales and molecular modalities are lacking. In this study, we generated multiplexed protein maps over a retinal organoid time course and primary adult human retinal tissue. We developed a toolkit to visualize progenitor and neuron location, the spatial arrangements of extracellular and subcellular components and global patterning in each organoid and primary tissue. In addition, we generated a single-cell transcriptome and chromatin accessibility timecourse dataset and inferred a gene regulatory network underlying organoid development. We integrated genomic data with spatially segmented nuclei into a multimodal atlas to explore organoid patterning and retinal ganglion cell (RGC) spatial neighborhoods, highlighting pathways involved in RGC cell death and showing that mosaic genetic perturbations in retinal organoids provide insight into cell fate regulation.

## Abstract (web summary)

A multimodal atlas of retinal organoid development shows spatial interactions over time.

## Main

Technologies to measure multiple molecular modalities in single cells are transforming our ability to explore developmental biology^1,2^. Transcriptomes and accessible chromatin can be profiled in thousands of cells per experiment^1,2^, and multiplexed imaging methods provide high-information-content spatial registrations of tissues^3^. Within developing systems, single-cell sequencing and image-based measurements can be used to reconstruct cell state trajectories, which promise new insights into the differentiation dynamics across lineages and spatial domains. Applied to human stem cell-derived organoids^4,5^, these technologies could be used to understand how molecularly defined cell states relate to tissue structure and morphological development and ultimately to create predictive virtual models of human disease. Indeed, it is a major goal in systems biology to generate in silico tissue models that incorporate increasing complexity, capturing multiple high-resolution length scales and including multiple cellular feature modalities^6,7^. A major challenge to achieve this goal is to integrate multimodal measurements across spatial scales in meaningful ways to reveal the mechanisms that drive tissue development and morphogenesis. In particular for human development, where embryonic samples are scarce, there is the additional challenge to achieve this with organoid models, which often lack stereotypic organization, with substantial heterogeneity within and between organoids. There is, thus, a large unmet need to implement and integrate multimodal technologies in developmentally dynamic and human-relevant model systems^8^.

Retinal organoids generated from induced pluripotent stem cells (iPSCs) offer an inroad into studying human retina development, identifying mechanisms of disease and facilitating the discovery of new treatments^9^. From the breakthrough discovery that optic cups spontaneously self-organize in three-dimensional PSC-derived cultures in vitro^10–12^, multiple different protocols to generate human retinal organoids have been developed^13–15^. Retinal organoids are composed of diverse neural cell types: rod and cone photoreceptors (PRs), bipolar cells (BCs), horizontal cells (HCs), amacrine cells (ACs), retinal ganglion cells (RGCs), non-neural Müller glia (MG) and retinal pigment epithelium (RPE). These cells self-organize into a stereotypical laminar structure with outer and inner plexiform layers and outer (PR nuclei) and inner (ACs, BCs and HCs) nuclear layers (ONL and INL), together with RGCs in the RGC layer. Remarkably, immunohistochemical analysis and single-cell transcriptome characterization of developed organoids have confirmed their high similarity to the primary human retina^14,16,17^, providing a foundation for understanding human retina development and disease in vitro. Retinal organoids develop over the course of months, reaching a maximal in vitro maturation state around the age of 30 weeks with current methods^14^. Multiple studies have shown the potential value of retinal organoids for modeling human vision disorders^18–21^. However, differentiation trajectories and morphological heterogeneity have yet to be quantitatively evaluated, and a reconstruction of the gene regulatory networks (GRNs) that underlie differentiation of each neuronal and glia cell type within the organoid tissues is lacking. More generally, we lack a foundation to integrate high-resolution and high-information-content imaging data of tissue organization with sequencing data to assess phenotypes within these complex organoid tissues.

In this study, we established an experimental pipeline for performing iterative indirect immunofluorescence imaging (4i)^22^ on histological sections of retinal organoids at high spatial resolution, and we developed a computational approach for inferring spatial developmental dynamics from multiplexed protein maps of heterogeneous organoids. We combined this with a dense single-cell RNA sequencing (scRNA-seq) and single-cell assay for transposase-accessible chromatin using sequencing (scATAC-seq) dataset covering 6–46 weeks of development, which can reconstruct differentiation trajectories into each of the major neuronal and glial lineages. Integration of all data modalities provides a first-of-its-kind digital representation of human retinal organoid development. The digital organoid map can be used to explore spatial interactions over time and predict gene regulatory modules underlying retinal neurogenesis. We performed follow-up ‘in organoid’ mosaic perturbations of selected transcription factors (TFs) and focused on the orthodenticle homeobox 2 (OTX2) regulon required for human retinal neurogenesis^23–25^. Altogether, we think that our work is a major advance toward a virtual human retinal organoid and our approaches should be adaptable to other developing organoid or other model systems.

## Results

### Spatial protein map of human retinal organoids and adult retina

**Fig. 1:**
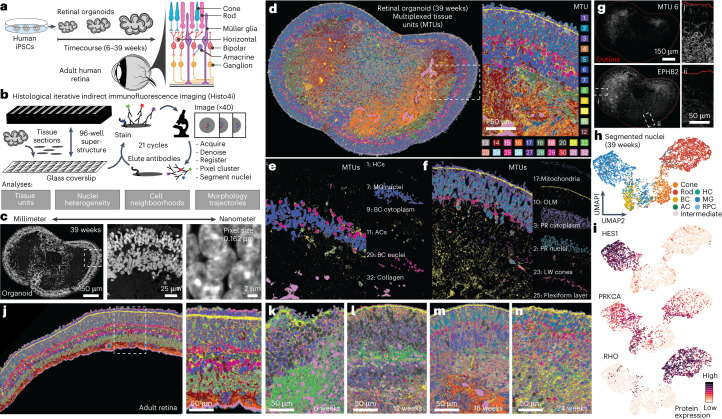
Highly multiplexed immunohistochemistry reveals scale-crossing features of developing retinal organoids and primary human retina. **a**, 4i was performed on tissues of a timecourse of retinal organoid development (6, 12, 18, 24 and 39 weeks) and adult retina tissue. Schematic shows retinal cell type organization. **b**, Schematic of the 4i methodology and analyses. FFPE tissue sections were placed on a 96-well format coverslip that was subsequently attached to a 96-well superstructure allowing immunohistological treatment, followed by imaging and antibody elution. Here, we performed 21 immunohistochemical staining cycles. Images were acquired at ×40 magnification with a high-NA silicone oil objective, tiled across the tissue. **c**, Representative 4i dataset image showing overview of a Hoechst stain of a 39-week organoid section and progressive magnifications from millimeter to nanometer scales. **d**–**f**, Example of pixel clustering of a 39-week organoid section. **d**, Thirty-two global MTUs resolve the tissue structure with image quality and label biological structures in individual samples. **e**,**f**, Biological structures identified by unique MTUs include HCs, BC cytoplasm and nuclei, ACs and structural elements such as collagen-rich areas (**e**) and peripherally located structures such as mitochondria, the OLM, PR cytoplasm and nuclei, long-wave (LW) cones and the plexiform layers (**f**). **g**, MTU 6 (top) is enriched for EPHB2 protein expression (bottom) and non-uniformly distributed in the organoid section (outlined by red line). Insets show regions with high (i) and low (ii) detection of EPHB2 fluorescence immunohistochemical signal. Patterned EPHB2 staining was observed in all 39-week replicates (11 sections and three organoids). **h**, Heterogeneity analysis of nuclei with UMAP projection based on protein features and colored by labels transferred from sequenced cells. All major types are identified, including RGCs, HCs, cones, ACs, rods, BCs and MG. **i**, Feature plots highlighting median signal level per nucleus of HES1, identifying nuclei located in the INL; PRKCA, enriched in BCs; and RHO, identifying cells located in the ONL. **j**–**n**, Primary adult human retina section (**j**) and retinal organoid sections from different timepoints (**k**–**n**) with pixels labeled by MTUs derived from communal clustering and comparable across samples.

To establish a spatial retinal organoid reference map, we applied 4i covering a timecourse of human retinal organoid development (6, 12, 18, 24 and 39 weeks; 2–4 organoids per timepoint; Supplementary Table 1) as well as an adult primary human retina (Fig. 1a). For each retinal organoid and the primary adult tissue, we performed multiplexed immunohistochemistry on 3-μm-thick formalin-fixed, paraffin-embedded (FFPE) sections (1–4 sections per sample; Supplementary Table 1 and Fig. 1b). We generated tiled images at ×40 magnification with a high-numerical-aperture (NA) silicone oil objective to cover length scales from the millimeter to the nanometer scale (pixel size, 0.1625 μm) (Fig. 1c). We established a panel of 63 antibodies covering major retinal cell types, subcellular compartments, morphological structures and signaling pathways split into three color channels (Supplementary Table 1). We obtained strong and reproducible signals over 21 imaging cycles, with usable data from 53 antibodies and a nuclei stain (Supplementary Fig. 1a,b). To achieve multiplexing, we aligned images^26^ across all cycles, allowing simultaneous display of any protein stain (Supplementary Figs. 1c–f and 2a,b). In our experimental design, we interspersed unstained cycles to subtract cycle-specific backgrounds, permuted antibodies to assess influence of antibody order and performed elution controls (Methods). These technical assessments confirmed that the staining patterns passing quality control were robust (Supplementary Fig. 2c–f). Altogether, this resulted in an expression matrix with over 400 million pixels, registering 53 immunofluorescence intensity measurements and a Hoechst stain from a retinal organoid developmental timecourse comprising 41 retinal organoid sections and an adult human retina sample.

For all tissues and timepoints, we established a multiscale analysis pipeline that includes an unsupervised machine-learning-based clustering of pixels from their 54-plex intensity profile (termed multiplexed tissue units (MTUs))^22^, nuclei segmentation (~330,000 nuclei) and assessment of nuclei heterogeneity and spatial arrangement from protein intensities and MTU distributions (Methods). MTUs can be generated for single samples or for the entire timecourse (global MTUs; Methods and Supplementary Fig. 3a–f). Global MTU analysis revealed 32 MTUs, which we analyze throughout the manuscript. Focusing on an exemplary 39-week organoid, these MTUs provide a detailed characterization of the spatial organization of the tissue (Fig. 1d). Hierarchical clustering and heat map visualization of average protein expression within each MTU highlight how each protein stain associates with each MTU (Supplementary Fig. 4a). We found that the MTU patterns are similar across different sections from the same organoid as well as between organoids of the same timepoint (Supplementary Fig. 4b,c). This indicates that MTUs provide a meaningful approach to quantify principles of tissue organization and composition in an unbiased manner, which is robust to the morphological heterogeneity observed within and between organoids. We observed that certain MTUs distinguish different cell types, whereas others resolve subcellular and tissue structures (Fig. 1e,f), illustrating their versatile multiscale nature. For example, MTU 9 and MTU 29 segment a subset of BC nuclei and cytoplasm, respectively; MTU 17 resolves mitochondria; MTU 10 highlights the outer limiting membrane (OLM); and MTU 25 labels the outer and inner plexiform layers. MTU 6 identified a surprising feature marked by ephrin type B receptor 2 (EPHB2) (Fig. 1g). EPHB2 functions in axon guidance and is asymmetrically expressed in retinal neurons demarcating dorsal and ventral domains of the developing mammalian retina^27^, suggesting that dorsoventral patterning domains can emerge during human retinal organoid development.

Focusing on segmented nuclei in an exemplary 39-week organoid section, variance analysis in the protein expression feature space and visualization in a uniform manifold approximation and projection (UMAP) embedding revealed eight molecularly distinct nuclei clusters (Supplementary Fig. 4d,e), and comparison of protein expression with single-cell transcriptomes^14^ enabled cell type assignment to each nuclei cluster (Fig. 1h,i and Supplementary Fig. 4e,f). The major retina cell types could be identified, including PRs, RGCs, HCs, ACs, BCs and MG (Fig. 1h). When combining all 39-week organoids into a combined embedding, we identified the same cell types distributed across all samples (Supplementary Fig. 4g).

Over the timecourse and in the adult primary tissue, we also observed striking progression of MTU and nuclei organization patterns, further showcasing the robustness of the methods and the scale-crossing richness of the dataset for exploring developmental phenomena (Fig. 1j–n and Supplementary Fig. 3). We provide a web application (EyeSee4is, https://eyesee4is.ethz.ch/) to facilitate access to the imaging data and computed features over the timecourse. Altogether, these data establish 4i on tissues as a flexible, robust, sensitive and high-dimensional method to describe organoid cell composition and structure based on protein measurements.

### Spatiotemporal dynamics of retinal layer formation

**Fig. 2:**
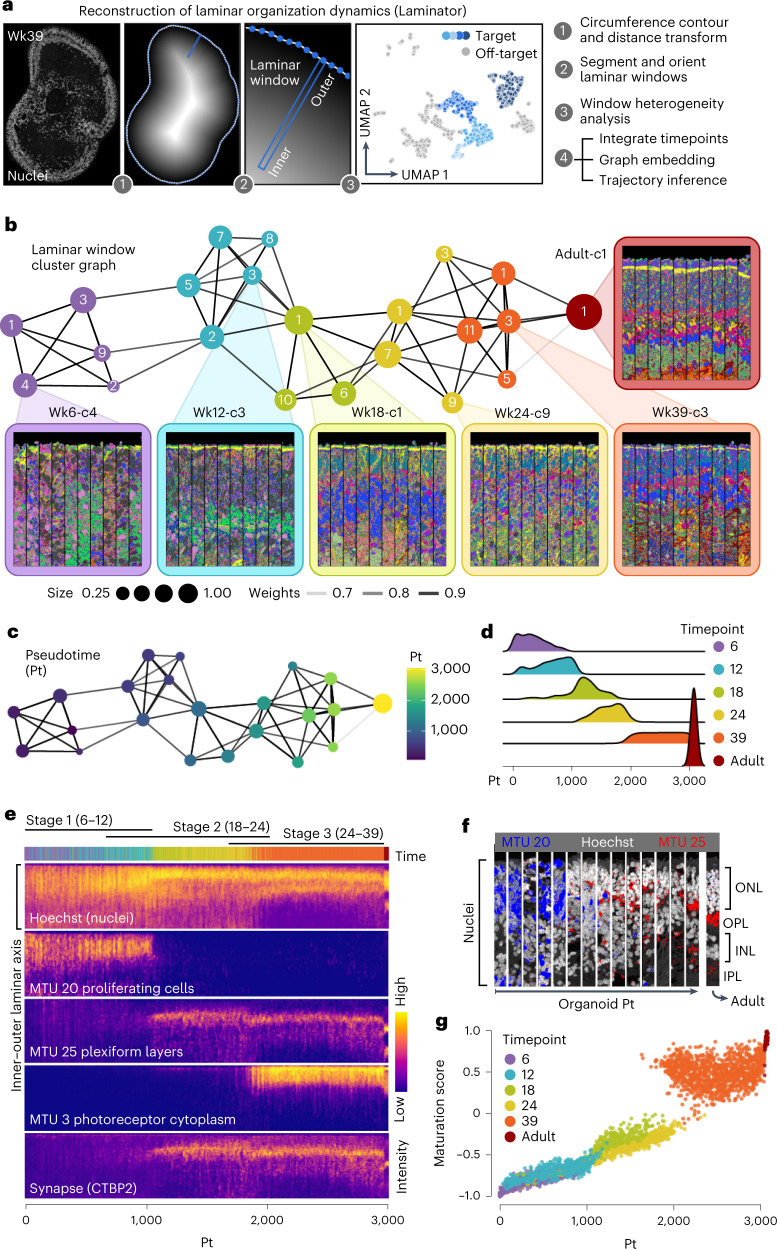
Analysis of laminar structure enables trajectory reconstruction and illuminates spatiotemporal dynamics of retinal organoid formation. **a**, Schematic overview of the Laminator algorithm developed for laminar window segmentation, vertical orientation and trajectory inference. Laminar windows measure 16.25 × 1.625 μm and are oriented on the tissue contour by maximizing the Euclidean distance transform of the masked organoid for each window. **b**, Force-directed graph embedding of laminar window clusters (numbered) colored by timepoint. Node size represents the fraction of laminar windows within a timepoint. Insets colored by timepoints show representative oriented laminar windows per cluster. **c**, Graph with laminar window clusters colored based on pseudotime from diffusion analysis. **d**, Density plot showing laminar window proportion along the pseudotime, grouped by timepoint. **e**, Heat map showing fluorescence intensity measurements (Hoechst and CTBP2) or MTU intensity profiles (MTU 20, MTU 25 and MTU 3) along the inner–outer laminar axis across oriented laminar windows ordered by pseudotime. **f**, Representative oriented laminar windows from multiple positions along the pseudotime course showing nuclei location (Hoechst, white), proliferating cells (MTU 20, blue) and plexiform layers (MTU 25, red) along the inner–outer laminar axis. **g**, Scatter plot showing signal similarity of each laminar window to adult laminar windows over pseudotime. Dots are colored by timepoint. c, cluster; Wk, week.

To study how the laminar structure in retinal tissue emerges, we developed a computational approach to reconstruct organoid laminar structure dynamics from multiplexed imaging data. This method (Laminator) establishes a contour around the organoid; segments and vertically orients adjacent laminar windows circumference-spanning each organoid; quantifies signals across laminar windows; analyzes laminar window heterogeneity; and applies graph embedding and diffusion analysis for trajectory reconstruction (Fig. 2a). We measured immunofluorescence intensities, MTU distributions and nuclei features in an inner-to-outer axis per laminar window, clustered the features per oriented laminar window and visualized relationships within and across organoids using a UMAP embedding (Supplementary Fig. 5a–c). From this analysis, we could distinguish various structural components of the tissue, such as highly organized nuclear layers, disorganized zones, non-retinal regions and aggregates of RPE. We selected retinal regions and used a force-directed graph to analyze the relationships between clusters across the timepoints (Fig. 2b) and applied diffusion analysis to establish a trajectory of retinal neuron layer development (Fig. 2c and Supplementary Fig. 5d,e). The reconstructed pseudo-temporal trajectory reflected a temporal progression (Fig. 2d) and enabled us to observe several interesting aspects of human retinal organoid development preceding retinal lamination and spanning PR maturation (Fig. 2e and Supplementary Fig. 5f–h). In early phases of the trajectory, there were abundant cells in the G2/M phase of the cell cycle marked by MKI67 and PCNA, associated nuclei localized to outer surfaces and exhibited elongated shapes (Fig. 2e and Supplementary Fig. 6a). Progenitor cells begin to differentiate into retinal neurons, and, subsequently, the plexiform layers emerge, becoming stratified between nuclei layers (Fig. 2e,f). In a later stage, PRs develop, and differentiation of neuronal and glial cells is established (Supplementary Figs. 5h and 6b,c). Overall, there is a clear and consistent pattern across expression and morphological signals that retinal organoids increase in similarity to the primary retina organization over the timecourse (Fig. 2g), consistent with scRNA-seq data^14^. We note that this reconstruction can be used to analyze the dynamic location of subcellular structures, such as mitochondria and P-bodies, and tissue structures, such as collagen and axonal fibers (Supplementary Figs. 5h and 6b,c). Altogether, these data provide a high-information-content spatial representation from protein measurements of human retinal organoid laminar developmental dynamics.

### Single-cell multiome analysis of human retinal organoids

**Fig. 3:**
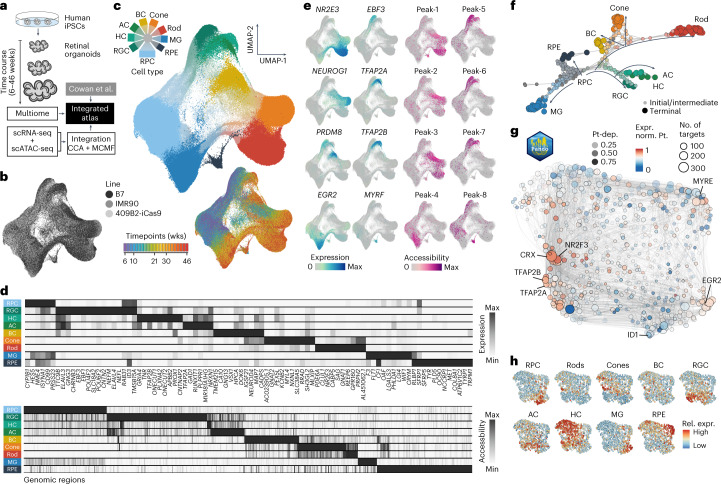
Timecourse single-cell multiomic data identify GRNs underlying human retinal organoid development. **a**, Paired scRNA-seq and scATAC-seq were performed on a timecourse of retinal organoid development. Multiome data were also acquired and used to assist with data integration. Together with previously published scRNA-seq data^14^, scRNA-seq and scATAC-seq data were combined into metacell representations containing both modalities using CCA and MCMF. **b**,**c**, UMAP embedding of metacells colored by iPSC line (**b**) or by annotated cell type (**c**, top) and timepoint (**c**, bottom). **d**, Heat maps showing average expression of representative marker genes (top) or chromatin accessibility (bottom) for each major cell type. **e**, Feature plots showing cell type marker gene expression (left) or chromatin accessibility (right). **f**, Branch visualization in a force-directed layout, with circles representing high-resolution clusters, with both RNA and chromatin access features colored by assignment. **g**, UMAP embedding of the inferred TF network based on co-expression and inferred interaction strength between TFs. Color and size represent expression weighted pseudotime of TF regulator and PageRank centrality of each module. **h**, TF network colored by expression enrichment for different cell types. Exp. norm., expression normalized; Pt, pseudotime; Pt-dep., pseudotime dependency; Rel. expr., relative expression; wks, weeks.

To provide multiomic resolution to human retinal organoid developmental dynamics, we performed scRNA-seq and scATAC-seq from the same cell suspension across a timecourse (6–46 weeks) of human retinal organoid development (Fig. 3a). The dataset incorporates 22 timepoints from four human iPSC lines, including iPSC lines with stable integration of doxycycline-inducible Cas9 in the AAVS1 safe harbor locus (iCas9) and previously published scRNA-seq datasets^14^ (Fig. 3b, scRNA-seq (in total 212,781), scATAC-seq (in total 151,684), and Supplementary Table 2). We constructed ‘multiomic metacells’ containing information on both transcriptome and chromatin accessibility using minimum-cost, maximum-flow (MCMF) bipartite matching^28^ within canonical correlation analysis (CCA) space^29^ (Supplementary Fig. 7a). The metacells were integrated using cluster similarity spectrum (CSS)^30^, and the integrated data were visualized using a UMAP embedding (Fig. 3c). In addition, we performed multiome measurements from the same cell (10x Genomics) at key developmental timepoints (15 weeks and 36 weeks, 13,645 cells), incorporated the cells into the integration and used the multiome data to assess the overall integration (Fig. 3c and Supplementary Fig. 7b–k). Altogether, the integration revealed a continuous distribution of cell states through the timecourse, with rods, cones, BCs, ACs, HCs, RGCs, RPE and MG annotated (Fig. 3d). The high dimensionality of the data could be used to identify marker genes and gene regulatory regions for the different cell types (Fig. 3d,e and Supplementary Table 3). Indeed, the cell-type-specific gene regulatory regions overlap with cell-type-specific promoters and enhancers (Supplementary Fig. 8), which may be useful to design cell-type-specific drivers for gene therapy^31^. These data provide a high-resolution feature assessment of human retinal organoid development from multipotent progenitor states and highlight the protocol reproducibility across different iPSC lines.

We next reconstructed the cell and GRNs that underlie human retinal development. We used RNA velocity^32^ and CellRank^33^ to generate a terminal fate transition probability matrix based on transcriptomes, which we used to construct a differentiation graph of high-resolution metacell clusters and assign branch identities. The graph, presented by a force-directed layout, reveals diversification of retinal cell types over the organoid timecourse (Fig. 3f). We used Pando^34^ to infer sets of positively or negatively regulated target genes (gene modules) as well as regulatory genomic regions (regulatory modules) for each annotated TF (Fig. 3g) and visualized module expression in a UMAP embedding. Feature plots reveal groups of TFs that associate with the development of specific cell types (Fig. 3h), including RGC development (POU4F2 and ATOH7), HC/AC/BC differentiation (TFAP2A, TFAP2B and PRDM8) and PR diversification (rod NR2E3; cone NEUROG1; both CRX). Globally, this GRN shows that regulatory region accessibility and TF expression track with stages of retinal organoid development and segregate during neuron diversification.

### Integrated multimodal map of retinal neurogenesis

**Fig. 4:**
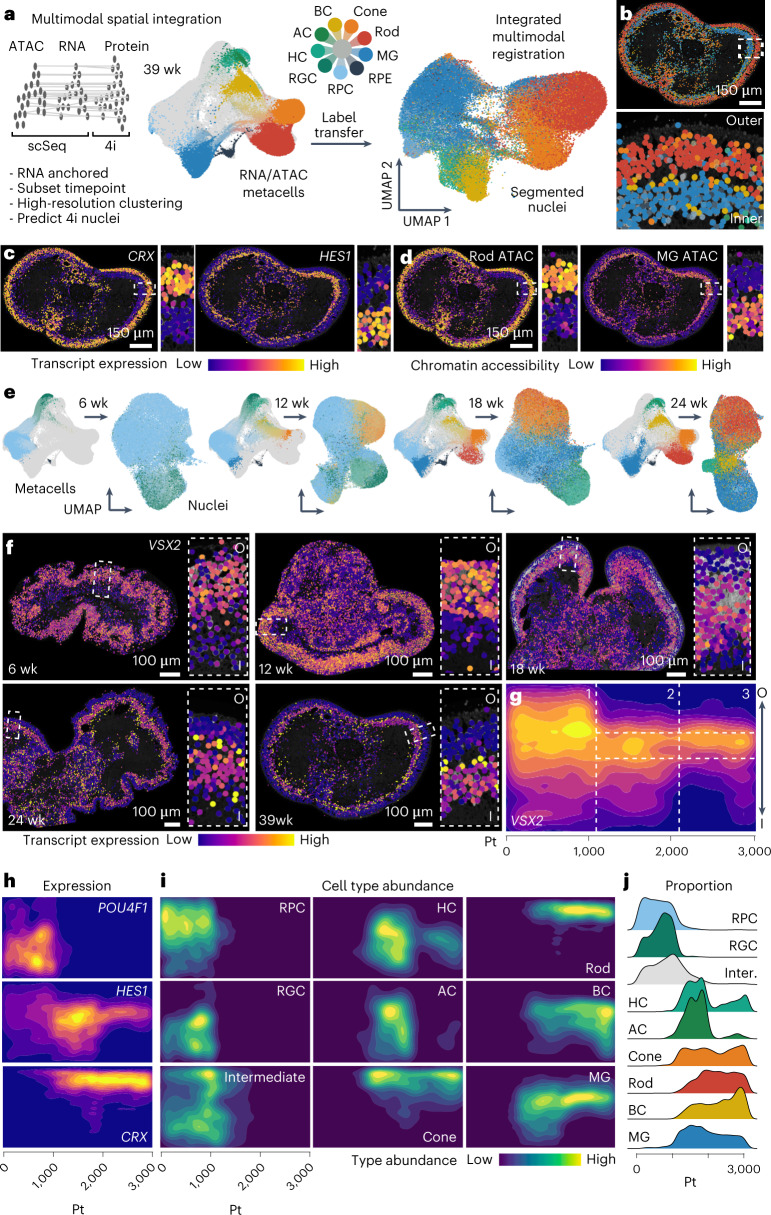
Multimodal integration provides a digital organoid representation of human retinal neurogenesis. **a**, Schematic for integrating accessible chromatin, transcriptome and protein modalities into spatially resolved and segmented nuclei. High-resolution clusters were generated from scSeq data (RNA/ATAC metacells) of the closest matching timepoints to the imaging data. Label transfer was predicted based on correlation of RNA and protein features in sequenced cells and imaged nuclei, respectively. Left UMAP shows timecourse metacell embedding based on transcriptome and colored by cell type with 39-week cells highlighted. Right UMAP shows nuclei embedding based on protein features colored according to label transfer from the transcriptome space. **b**, Overview and laminar zoom of a representative 39-week retinal organoid colored based on nuclei type assignment from the label transfer. **c**,**d**, Representative 39-week organoid nuclei colored based on RNA expression (**c**) or chromatin accessibility (**d**) of markers for PRs (*CRX*, **c**; chr17-81655189–81657223, **d**) or MG (*HES1*, **c**; chr11-126082119–126083088, **d**). **e**, Multimodal integration across the other timepoints. Left metacell embedding colored based on indicated timepoint. Right nuclei UMAP colored by label transfer from the transcriptome space. **f**, Timecourse retinal organoids colored based on *VSX2* transcript expression. Boxed inset shows zoom with inner (I) to outer (O) orientation. **g**, Heat map shows *VSX2* expression densities along the inner–outer and pseudotime axes. Dashed lines demarcate stages (vertical) and the INL (horizontal). 1, 2 and 3 refer to the organoid developmental stage as in Fig. 2e. **h**, Heat map showing expression of RGCs (*POU4F1*), MG (*HES1*) and PR (*CRX*) markers along the inner–outer and pseudotime axes. **i**, Heat map showing nuclei type abundance densities for the major annotated retinal organoid cell types/states along the inner–outer and pseudotime axes. **j**, Density plots showing proportion of each annotated nuclei type over the trajectory. Inter., intermediate; Pt, pseudotime; wk, weeks.

We next sought to integrate the sequencing and multiplexed imaging data to generate a multimodal spatial map of human retinal organoid development. We subsetted metacells from each sequencing timepoint, performed high-resolution clustering in the transcriptome space and predicted 4i nuclei type based on correlation between transcript and protein expression (Fig. 4a). In this way, the transcriptome and accessible chromatin modalities could be integrated with spatially localized nuclei in the 4i dataset, such that each nucleus contains chromatin, mRNA, protein and spatial features (multimodal nuclei; Fig. 4b and Supplementary Fig. 9a). Focusing on the developed organoid (39 weeks), we highlight *CRX* and *HES1* expression in PRs and MG, respectively (Fig. 4c), as well as rod and MG-specific gene regulatory regions (Fig. 4d). Applied to all sections and timepoints, we could map protein abundance, nuclei location, transcriptome and chromatin accessibility in all tissues, facilitating the spatial exploration of diverse data modalities across time (Fig. 4e and Supplementary Fig. 9). For example, we show that *VSX2* is initially highly expressed in progenitor cells localized toward the outer portion of the early organoid (6–12 weeks) and becomes restricted to BCs localized in the INL in developed organoids (39 weeks) (Fig. 4f,g). We could resolve the temporal emergence of each neuronal class, observing the transition from multipotent progenitor cells that were distributed throughout the lamina into intermediates and differentiated types that began to stratify over time to the INL and ONL (Fig. 4h,i). This analysis highlighted the emergence and disappearance of RGCs (marked by *POU4F1*) around weeks 6–18, a known deficiency of current retinal organoid protocols (Fig. 4h–j)^13,14,35^.

**Fig. 5:**
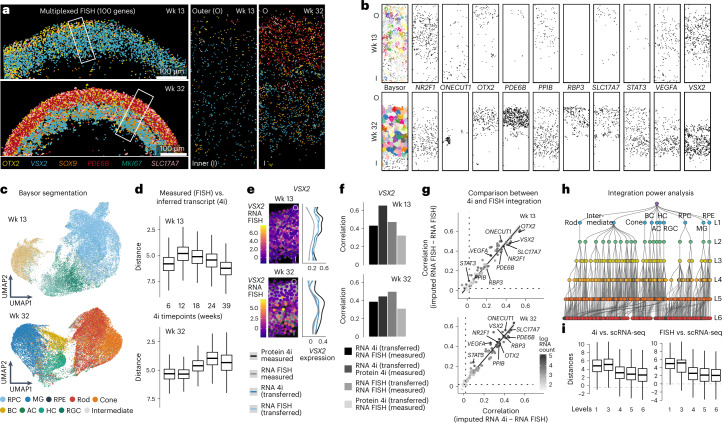
Multiplexed spatial transcript detection in retinal organoids enables evaluation of multimodal integration. **a**, Multiplexed RNA FISH in retinal organoid cryosections (week 13 and week 32). A six-transcript overlay (100 probed) is shown at two resolutions for one organoid at each timepoint. Scale bar, 100 µm; zoom, 55.2 × 165.6 µm. **b**, Transcript detection in a representative oriented (inner, I; outer, O) window for a week 13 (top) and a week 32 (bottom) organoid. First panel shows Baysor segmentation. **c**, Heterogeneity analysis of Baysor-segmented cell bodies from all organoid sections at week 13 (top) and week 32 (bottom). UMAP projection from FISH expression features and colored by labels transferred from sequenced cells. Major types are distinguished, including RGCs, HCs, cones, ACs, rods, BCs and MG. **d**, Box plots show averaged Euclidean genomic distances of matched spatial zones in week 13 (top, *n* = 1,697) and week 32 (bottom, *n* = 1,666) laminar windows from FISH data and transferred transcript expression in the multimodal integrated laminar windows from each 4i timepoint. **e**, Left, *VSX2* transcript detection in extended centroids of segmented cell bodies within an oriented week 13 (top) and week 32 (bottom) window. Right, line plot shows average *VSX2* expression across matched windows of measured protein (black) and RNA (gray) from 4i and FISH experiments and transferred RNA from integrated 4i nuclei (dark blue) and FISH cells (light blue). **f**, Bar plots show spatially constrained correlation within paired multimodal metacells (FISH and 4i) between transferred and measured RNA and measured protein and RNA. **g**, Scatter plot shows correlation between imputed and measured RNA within segmented features from FISH (*y* axis) and 4i (*x* axis) datasets. Genes (circles) are colored by transcript detection in the single-cell sequencing data. **h**, Power analysis comparing expression correlations in 4i and FISH transcriptome integrations. Clusters from scRNA-seq data were generated at different resolution levels. Major cell types were distinguished at level 1 (L1), with subsequent resolution describing biological and technical cell states. **i**, Box plots show distribution of distances calculated between the best and second-best matches for label transfer (Seurat) between scRNA-seq cells and 4i (left, *n* = 366,540) and FISH (right, *n* = 64,579) nuclei. The data suggest that both methods, on average, perform equivalently well and broadly resolve cell types. Wk, week.

To evaluate our findings with an orthogonal method, we assessed the transcriptomes represented within spatially resolved nuclei using multiplexed detection of RNA transcripts in situ (single-molecule fluorescence in situ hybridization (FISH); Molecular Cartography). We generated a highly resolved expression map probing 100 genes in retina organoids at 13 weeks and 32 weeks of development (total of 20 sections and eight organoids; Fig. 5, Supplementary Fig. 10 and Supplementary Table 7). Gene expression can be explored across the organoid section and within laminar windows (Fig. 5a,b and Supplementary Fig. 10a). We developed a pipeline to segment cell bodies, quantify transcripts within each nucleus and assess heterogeneity between nuclei based on gene expression, which resolved the major retinal cell types at each timepoint (Fig. 5c and Supplementary Fig. 10b–f). Using Laminator, we identified architectural variation within each organoid similar to what was observed in the protein expression space (Supplementary Fig. 11a–g). We compared the measured transcript expression within each retinal window to the predicted transcript expression in the integrated spatiotemporal reconstruction and found that most had the highest correspondence to windows within the nearest previously measured timepoint (Fig. 5d). We analyzed the correlation between measured and predicted transcript expression across laminar windows and found high correlation between most transcripts (Fig. 5e–g and Supplementary Fig. 11h). We also performed a power analysis to assess the ability to discern cell types using this integration. From the scRNA-seq data, we generated clusters at different levels of resolution (L1–L6). Notably, cluster level 1 distinguished each of the major retinal cell populations, and each subsequent cluster segmented each major cell type into further biological or technical states (Fig. 5h). We then compared inferred gene expression patterns in each multimodal integrated nucleus (containing RNA, chromatin access and protein information) with each cluster within the resolution hierarchy. We found that the integration is robust to at least the L3 hierarchy, and the 4i and FISH-based integrations perform similarly well (Fig. 5i). These data provide a multiplexed in situ map of retinal organoid transcripts at two timepoints, and the analysis supports a robust multimodal 4i and sequencing integration for identification of major cell types.

We used our multimodal integration to identify transcriptome features that associate with spatial features. We first focused on larger spatial domains. Previous analyses identified MTU 6 in a 39-week organoid that was distinguished by EPHB2 protein expression (Fig. 1g), a delineator of retinal dorsal–ventral patterning. We observed that multimodal nuclei in the integration also showed spatial heterogeneity in *EPHB2* transcript abundance (Supplementary Fig. 12a). We searched the multimodal nuclei for transcripts that spatially correlated and anti-correlated with *EPHB2* expression. This analysis identified gene sets enriched in nuclei in *EPHB2*-high and *EPHB2*-low domains, and these genes had high expression in MGs and BCs and lower expression in PRs (Supplementary Fig. 12b,c). We performed Gene Ontology (GO) term enrichment analysis of genes positively or negatively spatially correlated with *EPHB2*. This revealed general sensory or neuronal terms for genes negatively spatially correlated with *EPHB2*. In contrast, terms of genes positively spatially correlated with *EPHB2* related to metabolism and (axon) development, suggesting that cells high in *EPHB2* might be developmentally active and that some aspects of retinal axon pathfinding mechanisms can be detected in retinal organoids (Supplementary Table 4 and Supplementary Fig. 12d,e). These data support our previous observation that patterning domains can emerge in retinal organoids, which have an impact on longer-term expression patterns that emerge after weeks in culture.

We next used the integrated dataset to explore spatiotemporal features of cell type development, focusing on the emergence and loss of RGCs. We previously observed that RGCs emerge at 6 weeks, become abundant at 12 weeks, but are nearly absent at 18 weeks of development (Fig. 4). Nuclei in 6-week organoids could be ordered into a retinal precursor cell (RPC) to RGC differentiation trajectory based on either protein or RNA features (Supplementary Fig. 13a–d). This ordering revealed gene expression changes during differentiation (Supplementary Fig. 13e,f), and spatial analysis showed that RGCs accumulate at the organoid inner surface (Supplementary Fig. 13g), consistent with in vivo observations of RGC development^36^. To understand the loss of RGCs, we established a neighborhood analysis to explore microenvironmental variation in RGC locations. We segmented cell neighborhoods through a 6.5-μm radial extension from each RGC nuclei centroid, and then we searched for heterogeneity among neighborhoods based on annotated features (for example, MTUs, protein, RNA and chromatin access) (Supplementary Fig. 14a). Clustering and visualizing RGC neighborhoods from 12-week organoids based on MTU profiles in a UMAP embedding identified significant heterogeneity among the RGC neighborhoods (Supplementary Fig. 14b,c). Inspection of RGC neighborhoods on the images revealed differential location patterns between many of the clusters (Supplementary Fig. 14d). Interestingly, certain RGC neighborhoods were localized within the interior of the organoid, and nuclei within these neighborhood clusters exhibited cell death features, including intense Hoechst staining and nuclei fragmentation^37^, and other protein and MTU characteristics (Supplementary Fig. 14e–g). We explored heterogeneity in the transcriptome space and identified two major RGC types, distinguished by the presence and absence of *POU4F1* expression (Supplementary Fig. 14h). Subclustering revealed 11 clusters, and differential expression analysis between clusters highlighted cluster 6 as having a strong signature of apoptosis (Supplementary Fig. 14i,j). GO analysis revealed pathways and specific genes that may be involved in RGC preservation or induction of cell death, which have implications for human vision disorders, such as glaucoma^38,39^ (Supplementary Table 5). Altogether, these data and analyses showcase how the digital retinal multimodal map can be used to explore gene regulation and spatial feature co-variation, and cell neighborhood analyses can be performed for any cell type or spatial domain.

### Mosaic perturbation of TF regulomes

**Fig. 6:**
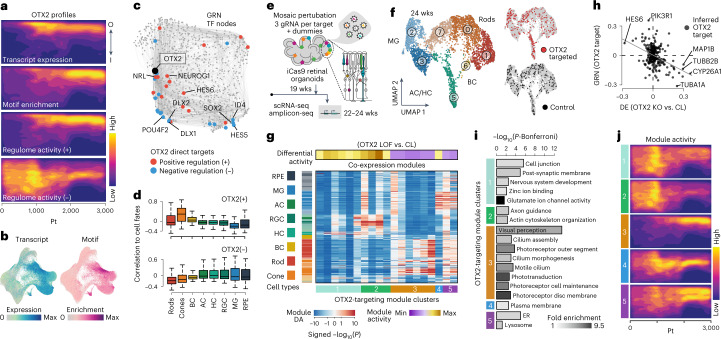
Mosaic genetic perturbation and scSeq in organoids highlights OTX2 regulome activity differences between retinal cell types. **a**, Heat map shows *OTX2* transcript expression, motif enrichment and positive regulome (+) and negative regulome (−) densities along the inner (I) to outer (O) and pseudotime axes from the reconstructed multimodal map. **b**, Transcriptome-based UMAP metacell plot showing *OTX2* expression (left) or motif enrichment within accessible chromatin peaks (right). **c**, Global TF GRN highlighting OTX2 (black node) and the predicted positively (red) and negatively (blue) regulated genes within the inferred OTX2 regulon. **d**, Box plots show the distribution of OTX2 positively (top, +) or negatively (bottom, −) regulated targets within the GRN based on the expression correlation of each target to different retinal cell fates. **e**, Schematic of single-cell perturbation experiment using the CROP-seq method. Three gRNAs targeting *OTX2* and four other TFs (Supplementary Information) were used together with a random non-targeting gRNA (dummy). Retinal organoids were infected with gRNA-containing lentiviruses at 19 weeks, and scRNA-seq and amplicon-seq were performed on suspensions at 22–24 weeks. **f**, UMAP projection colored by annotated cell type (left) or by cells with *OTX2* (top right, red) or dummy (bottom right, dark gray) gRNAs detected. Gray cells represent unknown or other targeted TFs. **g**, Heat map showing gene expression modules (columns) and their activity in cell clusters (rows). Left sidebar shows cluster type. Bottom sidebar shows module clusters. Top side bar shows the differential module activity for the *OTX2* gRNA cells relative to dummy control. **h**, Scatter plot showing the relationship between differential expression between *OTX2* LOF and control (*x* axis) and predicted directionality in the OTX2 GRN targets (*y* axis). **i**, GO enrichments for modules that are significantly affected by *OTX2* LOF. **j**, Heat map shows the module activity scores across the retinal organoid spatiotemporal multimodal map. CL, control; KO, knockout; Pt, pseudotime; wks, weeks; ER, endoplasmic reticulum.

The GRN analyses and integrated multimodal map illuminated TFs that are central regulators of development. To begin to understand how TFs regulate retinal cell type identity in human tissues, we established a pooled loss of function (LOF) experiment based on the CROP-seq protocol^34,40^ in developed retinal organoids (Supplementary Fig. 15a). We targeted five TFs (OTX2, NRL, CRX, VSX2 and PAX6) that are important for retinal development^41^ and are expressed dynamically over the organoid developmental timecourse (Supplementary Fig. 15b,c and Fig. 6a). We found that *OTX2*, *CRX* and *NRL* are expressed in rods, cones, BCs and the RPE (Supplementary Fig. 15c). *VSX2* and *PAX6* are expressed in RPCs, and their expression is maintained in BCs and RGCs/ACs/HCs, respectively (Supplementary Fig. 15c).

To perform the LOF experiment, we established an inducible Cas9 nuclease (409B2-iCas9) line and validated that it produces each of the major retina neuronal cell types (Fig. 3c,d). We generated a lentiviral library containing a GFP reporter^34^, targeting guides against *OTX2*, *NRL*, *CRX*, *VSX2* and *PAX6* and a non-targeting guide as control. The iCas9 line was used to generate retinal organoids, which were infected with the pooled lentiviral library at 19 weeks of development, to investigate the role of the TFs in a timepoint in which all cell types are already present. At 3–5 weeks after infection, GFP^+^ cells were sorted and used for scRNA-seq (10x Genomics) and target amplicon sequencing (Supplementary Fig. 15d,e). Based on RNA expression measurements, we generated a UMAP embedding, analyzed cell type heterogeneity and annotated the major cell types recovered in the experiment (Supplementary Fig. 15f). We performed expression module analysis^42^ and found that *OTX2* LOF cells showed the strongest effect, with OTX2 module genes being significantly misregulated (Supplementary Fig. 15g–i). We, therefore, focused subsequent detailed analyses on the effect of perturbation of the OTX2 regulon.

We first explored *OTX2* in our digital organoid map. The OTX2 regulon is distinguished by predicted positive regulation of genes enriched in rods, cones and BCs relative to the other retinal cell types, with the OTX2 motif being enriched within accessible chromatin of these cell types (Fig. 6a,b). Within the global GRN, we found that among direct and positively regulated OTX2 targets are *NRL*, *NEUROG1* and *HES6*, which are TFs that are enriched in rods, cones or BCs, respectively. Conversely, direct negative targets are *DLX1/2*, *HES5* and *POU4F2*, which are TFs enriched in fates that are predicted to be negatively regulated by OTX2 (Fig. 6c,d)^24^.

The CROP-seq experiment provided sufficient *OTX2* guide RNA (gRNA) detection across the different retinal cell types to assess the impact of predicted LOF on each cell type expression module (Fig. 6e–g). For *OTX2* LOF, we found that RGC, HC and AC expression modules were enriched and PR and BC expression modules were depleted in comparison to control cells (Fig. 6g). In addition, there was a correlation between GRN OTX2 target predicted regulation directionality and differentially expressed genes between *OTX2* LOF and control cells (Fig. 6h). The most depleted expression module in the *OTX2* LOF condition (module 3) had ontology enrichments for many aspects of visual perception, consistent with the role of OTX2 in maintaining PR identity and the critical role of OTX2 in the development and maintenance of the human visual system (Fig. 6i). Finally, we highlight the activity of these OTX2 regulated modules over the digital organoid map, showing that module 3 emerges temporally upon PR differentiation as they localize to outer positions within the lamina (Fig. 6j).

These findings are consistent with previous results in non-human model systems showing that OTX2 governs sister fate choices in the developing retina, particularly directing and maintaining PR and BC programs, while inhibiting the AC/HC/RGC programs^24,25,43^. In addition, we looked at how each targeted TF in the CROP-seq affects the regulome of the other TFs targeted in the CROP-seq (Supplementary Fig. 15j). Interestingly, this reveals that *OTX2* LOF induces a strong effect on the PAX6 (refs. ^24,25^) and CRX regulomes^25^. Because it is currently unknown how OTX2 controls *PAX6*, we used the GRN to predict this regulation and found that *PAX6* might be indirectly downregulated by OTX2 through DLX2 and POU2F2 (Supplementary Fig. 15k). Accordingly, DLX2 and POU2F2 regulomes are strongly affected by *OTX2* LOF (Supplementary Fig. 15l). Altogether, these data bring together spatiotemporal GRN analysis with perturbations using genetic manipulation to highlight the utility of organoids and digital multimodal maps to gain holistic insight into human retinal neurogenesis.

## Discussion

Organoid models of human physiology and pathophysiology are becoming important multicellular systems for basic and translational research. However, the field has lacked integrative experimental and computational approaches for phenotyping organoid development across spatial and temporal scales. Here we show that 4i on tissues combined with single-cell genomics can be a flexible and broadly applicable approach to generate high-information-content spatial and molecular descriptions of organoids and their primary counterparts covering chromatin, transcriptome and protein features. 4i on tissues is attractive as it uses off-the-shelf antibodies to measure protein expression spatially, and methods can be established for tissue processing, liquid handling and confocal imaging that provide data spanning subcellular, cellular and tissue scales. In a single experiment, we generated a 53-plexed protein map from 41 samples covering multiple organoid timepoints, crossing length scales of ~150 nm to several millimeters in a total of more than 400 million multiplexed measurements. We also generated a 100-plexed RNA transcript map from 20 samples with similar resolution. Sequencing and imaging data integration at this level of multiplexing can robustly resolve major retinal cell types using both the in situ protein and RNA measurements and resolve biologically meaningful cell states. Cell type and state resolution is dependent on antibody and RNA probe selection, which can be adapted per experiment. Our holistic analysis of organoid tissues provides data-driven approaches to explore global and local spatial heterogeneity. Thus, our multimodal map, together with previous assessments, suggests an optimistic view of the predictive capacity of retinal organoids.

Indeed, we provide evidence that the well-known master regulator OTX2 is required to maintain retinal neuronal identities, consistent with results on non-human models^24,25^. The vast majority of chromatin access, gene and protein expression profiles and cell differentiation profiles support the striking correspondence between organoid and primary retina counterparts. Human retinal organoids develop over many months in culture, and our data suggest that mosaic perturbation experiments can be performed at any point in development using inducible Cas9 iPSC lines to interrogate gene LOF. We also observed that organoids contain disorganized or malformed regions that are not often highlighted in the literature, and we developed methods that allow classification of tissue types to allow for targeted inclusion or exclusion for downstream analysis. By establishing a novel approach to assess intra-organoid and inter-organoid heterogeneity based on tissue segmentation and clustering, we can overcome barriers associated with organoid heterogeneity. Segmented tissue units can be arranged into trajectories to reconstruct morphological transitions (for example, lamination), and cell neighborhoods can be grouped to study microenvironmental variation associated with cellular states. We expect that these approaches will be useful for diverse organoid systems to explore tissue structure, to understand tissue developmental dynamics, to assess fidelity to primary counterparts and to quantify phenotypes in disease or other perturbation conditions.

Moving forward, a comprehensive organoid atlas will require integration of additional modalities together with temporal and spatial features. Future integrations may be achieved through incorporating ‘bridge’ measurements, such as combining 4i with measurements of protein and RNA or chromatin accessibility in the same cell (for example, CITE-seq^44^) or through combined multiplexed transcript and protein detection in the same or adjacent sections. Combined with experimental perturbations and models of disease, such virtual or digital organoid systems will allow for comparative analyses across organoid phenotype spaces that span molecular and tissue scales. We envision that such data-rich representations of organoid phenotypic landscapes will provide new insights into human biology and disease.

In conclusion, we demonstrate that 4i on developing organoid tissues is a robust, reproducible and high-resolution method to profile heterogeneity across many samples and scales. The method is capable of resolving cell types, morphological structures, organelles and protein localization, thereby providing analytical access to spatial features beyond gene expression. We developed Laminator as a tool to classify stereotypic tissue windows and reconstruct tissue developmental dynamics from timecourse multiplexed image data. We show that multiplexed protein maps and single-cell transcriptome and chromatin data can be integrated, and this phenotyping approach, especially across a developmental timecourse, can provide powerful multimodal information about cell type differentiation within a microenvironment. We provide a computational pipeline for 4i analysis and multimodal integration that can be adopted by the research community.

## Methods

### Primary human tissue samples

The human retina tissue was obtained from an anonymous multi-organ donor by sampling non-transplantable eye tissue that was removed in the course of cornea harvesting for transplantation^14^. The donor had no documented history of eye disease, and the personal identifier was anonymized before processing. All tissue samples were obtained in accordance with the tenets of the Declaration of Helsinki, and experimental protocols were approved by the local ethics committee. The sample included in this study is designated by the ID R-00646_06.

### Stem cell and organoid culture

Four human iPSC lines were used: B7 (01F49i-N-B7 (ref. ^14^)), IMR90 (iPS (IMR90)-4-DL-01, WiCell), B7-iCas9 (01F49i-N-B7-iCas9) and 409B2-iCas9 (ref. ^45^). B7 iCas9 carried integration of the construct pAAVS1-ieCas9 (ref. ^46^) in AAVS1 locus. For 4i experiments, B7 organoids at 6, 12, 18, 24 and 39 weeks were used. iPSCs were cultured in mTeSR1 (STEMCELL Technologies, 85857) or mTeSR Plus (STEMCELL Technologies, 100-0276) supplemented with penicillin–streptomycin (1:200, Gibco, 15140122) on Matrigel-coated plates (Corning, 354277). Cells were split 1–2 times per week after dissociation with EDTA in DPBS (0.5 mM) (Gibco, 12605010). Media were supplemented with rho-associated protein kinase (ROCK) inhibitor Y-27632 (5 μM, STEMCELL Technologies, 72302) after thawing. Cells tested negative by polymerase chain reaction (PCR) for mycoplasma infection (Venor GeM Classic, Minerva Biolabs). All cell lines showed normal karyotypes upon generation; the 409B2-iCas9 line acquired a common stem cell abnormality over time^45^; and the B7-iCas9 acquired a duplication in the centromeric region of long arm of chr20: q11.21–q11.22 (a known adaptation to cell culture growth conditions). All organoids were generated using the AMASS protocol^14^. To generate retinal organoids from the B7 and IMR90 lines, 600 cells were seeded in each microwell of an agarose chamber (MicroTissues 3D Petri Dish micro-mold, Z764019). 409B2-iCas9 organoids were generated with 1,500 cells per microwell and treated with BMP4 (55 ng ml^−1^, Bio-Techne, 314-BP) on day 6 with half medium change on days 9, 12 and 15 (ref. ^47^). For the multiplexed FISH experiment, 300 cells were used for each organoid.

### 4i sample preparation

Retinal organoids were embedded in 1% low melting agarose at ~37 °C. Embedded organoids were fixed in 4% paraformaldehyde (PFA) in PBS at 4 °C overnight. Primary human retina samples were fixed in 4% PFA in PBS at 4 °C overnight without agarose embedding. Post-fixation samples were transferred to 70% ethanol for at least 30 min before paraffin embedding. A 96-well format cover slip (NEXTERION coverslips, 1535661) was coated with poly-l-lysine (Sigma-Aldrich, P4832) for 1 h at room temperature. Tissues were microtome-sectioned (3 μm) and transferred to the coated 96-well coverslip and then incubated at 37 °C for at least 1 h before deparaffinization (60° for 45 min, 2 ×3 min Neo-Clear (Merck), 2 ×3 min 100% ethanol, 1 ×3 min 96% ethanol, 1 ×70% ethanol, 1 ×5 min ddH_2_O). Samples were then fixed in 4% PFA in PBS for 15 min at room temperature. For antigen retrieval, the sample plate was transferred to 800 ml of 1 mM citrate buffer (pH 6.0, 0.05% Tween 20, sterile filtered) and heated to 100 °C in a microwave (Milestone Micromed T/T Mega) for 20 min. Samples in the citrate buffer cooled at approximately room temperature for ~2 h with an open lid. The cover glass was removed from the buffer and patted dry between the samples with a tissue paper wrapped around a glass slide. Care was taken that the samples never dried out. The cover glass was then attached to the bottom of a 96-well self-adhesive super structure (Merck, GBL204969), placing each sample into a well, and wells were filled with PBS until further processing.

### Multiplexed single-molecule FISH sample preparation

Retinal organoids (week 13 and week 32, cell line B7, four organoids per timepoint) were fixed in PAXgene Tissue FIX (Qiagen, 765312) for 2 h at room temperature and stabilized for 2 h in PAXgene Tissue STABILIZER (Qiagen, 765512). Organoids were allowed to sink O/N in 30% sucrose in PBS (w/v) solution at 4 °C, embedded in OCT and frozen in 2-methylbutane (Sigma-Aldrich, 106056) on dry ice and stored at −80 °C. Organoids were cryosectioned (10 µm), and slices were placed within the capture areas of cold glass slides provided by Resolve Biosciences. Samples were transported on dry ice for analysis, whereby tissue sections were thawed and washed at room temperature in isopropanol, 95% ethanol and 70% ethanol. Fixed samples were used for Molecular Cartography (100-plex combinatorial single-molecule FISH (smFISH)) according to the manufacturer’s instructions (protocol 1.3). In brief, tissues were treated with buffer DST1, primed for 30 min at 37 °C and hybridized overnight with gene probes. Samples were washed and fluorescently tagged for imaging.

### Multiplexed smFISH probe design

The 100-gene panel (Supplementary Table 7) was selected using geneBasisR^48^, given the 4i gene panel and the top five marker genes of major cell types as the pre-selected genes. Genes with transcripts per million (TPM) > 10,000 in any major cell type were excluded. Probes were designed using the highest-scoring probes from Resolve’s proprietary algorithm based on full-length protein-coding transcript sequences from the ENSEMBL database (GENCODE annotation tag ‘basic’)^49,50^. To filter highly repetitive regions, the abundance of k-mers was obtained from the background transcriptome using Jellyfish^51^. Target sequences were scanned for all k-mers, with preference to rare k-mers for probe design. A probe candidate was generated by extending seed sequences to a threshold hybridization stability, and probes were filtered through assessing mapping to a background transcriptome using ThermonucleotideBLAST^52^. Specific probes were then scored based on the number of on-target matches (isoforms), which were weighted by their associated APPRIS level^53^, favoring principal isoforms and those with protein-coding binding sites.

### Multiplexed smFISH imaging

Imaging was performed on a Zeiss Celldiscoverer 7 at ×25 magnification, with the ×50 Plan Apochromat water immersion objective, NA of 1.2 and a CD7 CMOS camera (Zeiss Axiocam Mono 712, 3.45-µm pixel size). Image settings were optimized for each signal, 1,000-ms exposure per FISH channel and 20 ms for DAPI. *z*-stacks were recorded for each channel according to the Nyquist–Shannon principle. Two channels were imaged in parallel. Eight imaging rounds were performed to obtain 16 *z*-stacks per sample. Automation of the staining and imaging cycles was achieved using a Python script using the scripting API of the Zeiss ZEN software. For improved nuclei segmentation, DAPI was reimaged after the experiment with conditions identical to the 4i imaging.

### 4i imaging

Iterative staining and elution cycles^22^ included Hoechst (Invitrogen, 33342) to the secondary antibody staining solution at a dilution of 1:500. Pipetting was automated on a Hamilton STAR robotic system. Secondary antibodies were applied at a dilution of 1:500. Primary antibodies and dilutions used can be found in Supplementary Table 1. Imaging was performed using a Nikon Ti2 inverted microscope with a Yokogawa CSU- W1 SoRa spinning disk add-on and an ORCA-Flash4.0 Digital CMOS camera, C11440, and NIS-Elements software. A ×40 magnification was achieved using the Nikon PLAN APO ×40/1.25 SIL MRD73400 objective. The laser settings were kept constant throughout the experiment—20% UV (405 nm), 20% green (488 nm), 20% red (561 nm) and 40% far red (640 nm) with 100-ms exposure each. Each two channels, 405 nm and 561 nm or 488 nm and 640 nm, were acquired in parallel. Twenty percent of the camera sensors at each side were cropped to minimize shading effects and maximize stitching accuracy, and 10% overlap was used for stitching. Images were acquired as *z*-stacks with a total of 6-μm thickness and 0.5-μm distance between image planes. Every image consisted of a 6 × 6 tiling array. The image cycling strategy constituted ‘staining cycles’ (including elution and restaining) and ‘elution cycles’ (including elution and mock staining steps without addition of antibodies). The elution cycles were performed every six cycles, allowing assessment of background signal across the experiment (Supplementary Table 1). The elution cycles were used for background subtraction. In each staining cycle, three primary and secondary antibody combinations and a Hoechst stain were imaged (rabbit antibody, green, 488 nm; mouse antibody, red, 568 nm; third species antibody, far red, 647 nm; Supplementary Table 1). Certain protein stains were excluded by visual examination.

### Image pre-processing and registration

Background subtraction was achieved through acquiring a dark-field image across *z*-stacks and fluorescence settings identical to stained image acquisitions but with lasers off. For each channel, an average intensity projection per tile and an average of all tiles were generated, resulting in dark-field images. Sample image tiles were maximum intensity *z*-projected, and, for each channel, the dark-field images were subtracted from every tile, without shading correction. Stitching was performed after maximum intensity *z*-projection using multichannel images with a Fiji plugin^54^. Hoechst images were used to align images across all cycles using SimpleElastix^55^, and degradation of Hoechst staining required iterative image alignment (images cycle00 - cycle6 to cycle01, cycle07 - cycle11 to cycle06, cycle12 - cycle16 to cycle11, cycle17 - cycle21 to cycle16). A tissue mask from the Hoechst channel in the reference cycle facilitated alignment. Alignment was performed in two steps: a rigid transformation followed by an affine transformation. The parameters used for the SimpleElastix algorithm (Supplementary Table 6) were optimized by visual examination of the results. Images of every cycle and every sample were assessed for alignment accuracy. In cases in which alignment was suboptimal, different cycles were chosen as reference to improve the alignment.

### Image masking, denoising and background removal

ATP1A1 (membrane) and TUBA4A stains were used to generate tissue masks and isolate primary objects, by scaling images to the bottom 1st and upper 99th percentiles, divided by 2 and summed across images. Additionally, Otsu thresholding, dilation, expansion and closing of holes ensured that no tissue was excluded in tissue masking. All images were cropped to the bounding box based on these masks and denoised using the denoise_nl_means algorithm from scikit image in fast mode^56^. For denoising, a sigma was estimated from the images. Further parameters were patch_size = 5, patch_distance = 6, cutoff distance (h) = 0.8 × estimated sigma. Several stainings had high spurious signals in areas corresponding to collagen-labeled areas, which were subsetted and excluded (Supplementary Table 1). Elution cycles bracketing imaging cycles were used to support background removal. Elution cycle images were scaled to the upper and lower 1st percentiles, multiplied by the factor according to the desired proportion and summed. A fast non-negative least squares algorithm (https://github.com/jvendrow/fnnls) was used to gauge a factor with which to multiply the background model before subtracting it from the respective foreground image.

### Pixel matrix normalization

After registration, every sample has a defined pixel grid that is consistent across all cycles, and a matrix can be generated in which every row corresponds to a pixel and every column to a signal measurement. Imaging conditions were constant within every color channel throughout the experiment. To account for remaining signal divergences, samples were normalized while preserving divergent signals due to biological differences. All images were combined per timepoint to generate a pixel–signal matrix. For each timepoint, we calculated the mean and s.d. per stain channel, *z*-scored every image, multiplied it by the timepoint and color-channel-specific s.d. and added the timepoint and color-channel-specific mean (reverse *z*-scoring)^22^. Finally, every image channel was scaled to 0–1, where 0 is the bottom 1st percentile, and 1 is the upper 99th percentile across all images.

### MTUs

Pixel clustering^22^ was performed on normalized and scaled values per sample and globally across all samples. For global clustering, a normalized global pixel matrix was generated by appending a random subset of every sample matrix row-wise by a factor of 1,000, but, for the single-sample clustering, the process used a random subset only on an individual sample. In both cases, FlowSOM^57^ was used to generate a self-organized pixel map (30 × 30 grid, Euclidean distance, 10 runs). Median intensities of the fSOM output clusters were further clustered using phenograph^58^. The number of *k*-nearest neighbors specified for phenograph clustering was chosen by locating the inflection point (neighbor numbers versus number of clusters detected) using KneeLocator of the kneed package^59^. All pixels were assigned to the phenograph clusters by closest Euclidean distance in 4i intensity measurement space. MTU images were generated by placing cluster-assigned pixels into the masks outlining the organoid images and assigning colors to pixels according to the assigned cluster number.

### Nuclei segmentation and features

Nuclei were segmented using iterative optimization of Cellpose^60^ parameters based on Otsu thresholding of the Hoechst signal from the reference cycle images. The parameters used were model_type: cyto, diameter: 35, flow_threshold: 0.8, cellprob_threshold: 0 and channels: [0, 0]. Nuclei features were retrieved using the segmented nuclei, the normalized pixel matrix and the regionprops_table function of the scikit-image toolbox^56^. Fluorescent intensity values measured per stain are the bottom 5th and top 5th percentiles, median, mean and pixel count (sum of all intensity values per nucleus). Spatial features, radial distance and nuclei density were measured per nucleus. For radial distance, the refined mask was distance transformed, and the mean distance value per nucleus was measured. For nuclei density, an elliptical structuring element with 100 × 100 pixels^61^ was used as a kernel, and the number of nuclei in a kernel around the centerpoint of each nucleus was used as a nuclear density measurement. For visualization, nuclei features median fluorescence intensity values were scaled using the StandardScaler algorithm^62^, and a UMAP embedding^63^ was calculated from the first eight principal components (PCs) of the scaled features. Clustering was performed using the AgglomerativeClustering algorithm of the sklearn package^62^, and the number of clusters was set to 8 for clustering of a single replicate.

### Reconstruction of laminar organization dynamics

Contour outlines of all organoid samples and the adult primary sample were extracted using the skimage function find_contours()^56^ after applying a Gaussian filter (*σ* = 50) and Otsu thresholding to the respective mask images. Contour coordinates for the primary adult sample were filtered to mark the outer circumference. Distance transformations were generated using the ndimage function distance_transform_edt() on respective mask images and applying a Gaussian filter (*σ* = 25). The center of the outer edges of the laminar windows (100 × 1,000 pixels, 16.25 × 162.5 μm) were positioned on every 100th coordinate of the respective contour outlines and oriented along the inner–outer axis by iteratively rotating each window on their respective distance transformed image and maximizing the sum of captured distance transformation signal under each laminar window. Intensity profiles were extracted for all protein and MTU modalities by averaging across the inner–outer axis of each laminar window. Laminar windows positioned in straight contours and related to organoid regions outside of the field of view were removed, and windows with low signal or within damaged tissue were excluded by filtering windows for having more than 99% pixels with assigned MTUs. The laminar window intensity profiles of each protein and MTU modality were then reverse *z*-scored according to organoid section and timepoint and scaled between 0 and 1. Intensity profiles were smoothed by applying a one-dimensional (1D) mean filter (window size = 20) and downsampled by a factor of 2. Distances between laminar windows were calculated by fast Fourier transforming the intensity profiles and calculating the Euclidean distances of the first 10 frequency components for each feature using the TSDist package in R^64^. The resulting distance matrices were analyzed using the DistatisR method^65^. Laminar tissue heterogeneity was assessed by diffusion analysis^66^ of the log_10_(*x* + 1) transformed and across features aggregated distance matrix of laminar window intensity profiles. Results were then visualized by calculating UMAP embeddings for timepoint subsets of the first 10 diffusion components (DCs) and clustering laminar windows of each timepoint by performing Louvain clustering on the respective UMAP embeddings. Clustering performance and properties of laminar window clusters were evaluated by reconstructing the MTU images for all oriented laminar windows and ordered by timepoint-specific pseudotime. Louvain clusters were also mapped onto Hoechst stain images of each organoid, and clusters were selected for each timepoint not overlapping pigmented epithelium or other disorganized areas. All Louvain clusters from the primary tissue were retained and merged. A trajectory of laminar windows for the selected Louvain clusters (week 6: *n* = 5; week 12: *n* = 5; week 18: *n* = 3; week 24: *n* = 4; week 39: *n* = 4; adult: *n* = 1) were then reconstructed by applying diffusion pseudotime analysis.

A maturation score was calculated by measuring similarity of laminar windows to the start and endpoint of the trajectory. The averaged compromised distances (DistatisR) to the upper and lower 5% quantile of pseudo-temporal ordered laminar windows were calculated, subtracted from each other and scaled between −1 and 1. A constrained force-directed graph layout of laminar window Louvain clusters was generated by first filtering edges between clusters for being from the same or adjacent timepoints and then calculating edge weights by averaging the corresponding inter-laminar window cluster compromised distances to construct a *k*-nearest neighbors graph (*k* = 4) from the resulting adjacency matrix. Exemplary laminar window clusters for each timepoint were illustrated by randomly selecting 10 windows from each cluster and appending reconstructed MTU laminar windows along the pseudo-temporal trajectory. Spatiotemporal heat map visualization of features along the trajectory was achieved by scaling intensity profiles between the lower 1% and upper 5% quantiles and smoothened by position on the inner–outer axis along the pseudo-temporal axis by applying a 1D mean filter (window size = 15). Average heat map of protein and MTU intensities along the pseudotime trajectory were generated by averaging intensity profiles across the inner–outer axis and smoothed with a 1D mean filter (window size = 25) along the pseudo-temporal axis.

### scRNA-seq, scATAC-seq and multiome for the developmental timecourse

Retinal organoids were generated from four different stem cell lines (B7, IMR90.4, 409B2-iCas9 and B7-iCas9). Then, 1–3 organoids of the same batch were pooled and dissociated at multiple timepoints across the organoid developmental timecourse (Supplementary Table 2). Organoids were washed three times with HBSS without Ca^2+^/Mg^2+^ (STEMCELL Technologies, 37250). Single-cell suspensions were obtained using a papain-based dissociation kit (Miltenyi Biotec, 130-092-628)^14^. In brief, 1 ml of pre-warmed papain solution was added to the organoids and incubated for 10 min at 37 °C. To facilitate dissociation, the mix was pipette-mixed every 5 min with a p1000. Enzyme mix A was added and mixed by inversion and incubated for 10 min at 37 °C. Samples were pipette-mixed until tissue dissociation was confirmed via visual inspection. After incubation, cells were centrifuged for 5 min at 300*g* and 4 °C. Cells were resuspended in 250 μl of PBS + 0.04% BSA and sequentially filtered through a 70-μm filter (pluriSelect Mini, 43-10070-50) and a 40-μm filter (pluriSelect Mini, 43-10040-40). Cell counts were assessed with a trypan blue assay on the automated cell counter Countess (Thermo Fisher Scientific). For scATAC-seq, nuclei were isolated according to the protocol provided by 10x Genomics using the low input protocol and a lysis time of 3 min. Nuclei were loaded in a concentration that would result in a recovery of 10,000 nuclei. scATAC-seq libraries were generated with the Chromium Single Cell ATAC version 1.1 Library & Gel Bead Kit and sequenced on Illumina’s NovaSeq platform, NextSeq 550 or HiSeq 4000. For scRNA-seq, up to 8,000 cells were targeted, and scRNA-seq libraries were generated with the Chromium Single Cell 3′ version 3.1 Library & Gel Bead Kit. Libraries were pooled and sequenced on llumina’s NovaSeq platform, NextSeq 550 or HiSeq 4000. Combined scRNA-seq and scATAC-seq (multiome) were generated with the Chromium Single Cell Multiome ATAC + Gene Expression kit. Nuclei were isolated as described for scATAC-seq. The gene expression and accessibility libraries were FAB treated and sequenced on Illumina’s NovaSeq platform.

### Single-cell sequencing data pre-processing and multimodal data integration

For the scRNA-seq data of each retinal organoid sample, Cell Ranger (10x Genomics, version 4.0.0) with default parameters was used to align reads to the human reference (GRCh38, 10x Genomics, version 3.1.0) to generate the transcript count matrix for cells. Additional quality control was performed by excluding cells with detected transcript number fewer than 1,500 or higher than 20,000 as well as those with high mitochondrial transcript percentage (>20% for all the samples, except >40% for GB2_scRNAseq). The exonic and intronic count matrices were generated via dropEst^67^. For the scATAC-seq data of each retinal organoid sample, Cell Ranger ATAC (10x Genomics, version 1.2.0) with default parameters was used to align reads to the human reference (GRCh38, 10x Genomics, version 1.1.0) to generate the peak fragment count matrix for cells. Additional quality control was performed by excluding cells with detected ATAC fragments fewer than 200 or more than 10,000; cells with fewer than 20% fragments in the called peaks; cells with fragments at the blacklist genomic areas >2.5%; cells with nucleosome signal >3; and cells with transcriptional start site (TSS)<2.

To integrate scRNA-seq and scATAC-seq data measuring the same cell suspension of a retinal organoid sample, we modified a procedure^34^ as follows. Seurat (version 4.0.0) was used to normalize and log-transform the scRNA-seq data, identify highly variable genes (vst method, 3,000 genes, followed by excluding mitochondrial and ribosomal genes), data scaling, principal component analysis (PCA, select top 20 PCs) and Louvain clustering (resolution = 0.5). Average transcriptome profiles were calculated for each cluster. Signac (version 1.4.0) was used to normalize the scATAC-seq data (default settings); perform partial singular value decomposition (SVD) on the normalized TFIDF of peaks with fragment detected in more than 0.5% of cells to obtain the latent semantic indexing (LSI) representation; and Louvain clustering (resolution = 0.5) based on the 2nd to 30th LSI components. Gene activity scores of annotated genes in each cell of the scATAC-seq data were calculated using Signac, and average gene activity scores were obtained for each scATAC-seq cluster. For each scRNA-seq cluster, the scATAC-seq cluster with the highest correlation across scRNA-seq highly variable genes that have non-zero gene activity scores in the scATAC-seq data was identified, and vice versa, resulting in a bipartite nearest neighbor network of clusters that contains multiple cluster components that are disconnected from each other. Next, CCA, implemented in Seurat, was performed on the scRNA-seq data and the scATAC-seq data represented by the gene activity scores based on the anchoring features selected by Seurat (same number as the highly variable genes in the scRNA-seq data). An MCMF analysis was applied to cells from the two modalities in each cluster component. This constrained MCMF bipartite matching procedure resulted in bipartite edges, each of which connects one cell in the scRNA-seq data with one cell in the scATAC-seq data. For each cell in the scRNA-seq data with at least one cell in the scATAC-seq data paired, a bimodal metacell was formed, with the RNA component being the scRNA-seq data and the ATAC component being the union fragments of the paired scATAC-seq cell.

To evaluate the integration performance, an unweighted *k*-nearest neighbor (*k* = 20) graph was obtained for the scRNA-seq and scATAC-seq sample separately, based on Euclidean distances across the top 20 PCs for the scRNA-seq data and Euclidean distances across the 2nd to 30th LSI components for the scATAC-seq data. We defined the distance between two cells as the shortest distance on the *k*-nearest neighbor graph and then calculated the modal structure matching score (MSMS), which was defined as the Spearman correlation between the pairwise distances of cells in scRNA-seq with at least one cell in the scATAC-seq data paired and the pairwise distances of the paired cells in the scATAC-seq. A sample integration with MSMS < 0.1 was considered as a failure and was not used when forming the bimodal metacells.

For the multiome data, Cell Ranger ARC (10x Genomics, version 2.0.0) was used to align reads of both the RNA library and ATAC library to the human reference (GRCh38, 10x Genomics, version 2020-A-2.0.0) to generate both the transcript and peak fragment count matrices. Additional quality control was applied with varied criteria for the two samples, on the transcript count number, on the ATAC fragment number and on mitochondrial transcript percentages. To generate a unified peak list to combine the chromatin accessibility profiles in different retinal organoid samples, we grouped all the scATAC-seq data into four groups based on organoid ages (0–10 weeks, 11–20 weeks, 21–30 weeks and >30 weeks) and used MACS2 to perform peak calling on each group separately and merge (using the Signac-implemented wrapper function CallPeaks in default parameters). Based on the new peak list, the fragment number per peak of cells in the scATAC-seq and multiome data were requantified.

To integrate single-cell data across timepoints, we merged the scRNA-seq data (which contained ATAC-integrated metacells and cells without paired scATAC-seq data) and multiome data, together with a public scRNA-seq data of retinal organoids^14^. Focusing on the transcriptomic profiles, highly variable genes (vst method, 3,000 genes) were identified for each of the three datasets, and those identified in at least two datasets were considered as the overall highly variable genes. Data scaling (cell cycle scores regressed out) and PCA (top 20 PCs) were performed, and sample integration was performed using CSS^30^ stratified on samples (cluster resolution = 1). PCA (top 20 PCs) was applied to the resulting CSS matrix to generate the PCA-reduced CSS representation. Louvain clustering (resolution = 0.1) was applied, and one resulting cluster (cluster 6) was excluded for its expression of mesenchymal cell markers (for example, DCN). The same procedure was applied to the remaining cells, resulting in the new PCA-reduced CSS representation of the data. Louvain clustering (resolution = 0.5) was performed, and cells in three of the clusters were further excluded from the following analysis, for their expression of brain cell markers (for example, FOXG1 and GFAP). The UMAP embedding of the remaining cells was generated given their PCA-reduced CSS representation.

Cells of all the scATAC-seq data, as well as those of the multiome data, were merged and integrated, considering their accessibility profiles across the unified peak list, using CSS stratified on samples, after data normalization with TFIDF and generating LSI representation with SVD (2nd to 30th LSI components) using peaks detected in more than 0.5% of cells. PCA was performed on the resulting CSS matrix to get the PCA-reduced CSS representation (top 20 PCs). Harmony^68^ was also performed based on the 2nd to 30th LSI components with default parameters to generate a Harmony representation. Louvain clustering (resolution = 0.5) was performed based on the PCA-reduced CSS representation, and the clusters with enriched cells without any paired cells in the scRNA-seq data (Bonferroni-corrected one-sided Fisher exact test *P* < 0.01) were excluded. Next, the cluster labels of the scRNA-seq cells were transferred to the scATAC-seq data for those cells with only one paired cell in the scRNA-seq data or multiple paired cells but all sharing the same cluster label. For the rest of the cells, a network propagation procedure was developed to infer their corresponding cluster labels as follows. First, three unweighted *k*-nearest neighbor (*k* = 20) graphs were generated for the scATAC-seq cells, based on the LSI, PCA-reduced CSS and Harmony representation, respectively. The three graphs were averaged to form a weighted *k*-nearest neighbor graph. The adjacency matrix of the resulting *k*-nearest neighbor graph was obtained and row-normalized (that is, sum of each row is 1). The normalized adjacency matrix, denoted as the propagation matrix *P*, was further modified, so that, for any cell *i* that has a unique transferred cluster label from the scRNA-seq data, *P*_*i,i*_ = 1 and *P*_*i,j*_ = 0 if *i* ≠ *j*. This propagation matrix was then used to perform network propagation as *S*^*t*^ = *P* × *S*^*t*−1^, where *S*^0^ is the initial transferred cluster identity matrix: *S*^0^_*i,j*_ = 1 if cell *i* has a paired cell in the scRNA-seq data in cluster *j*, otherwise 0. The propagation was performed 100 times. The cluster with the highest propagated score was considered as the transferred cluster label of a given cell. The transferred-label-based cleanup was then performed by excluding cells with no paired cells in the scRNA-seq data as well as cells with transferred cluster labels matching the mesenchymal or brain clusters. Afterwards, the similar procedure of dimension reduction and data integration across samples was applied. A UMAP embedding was generated based on the PCA-reduced CSS representation.

### Cell type annotation and developmental trajectory characterization

Based on the combinatorial expression of canonical cell type markers in the mentioned Louvain clusters (resolution = 0.5), cells in the integrated retinal organoid cell atlas were coarsely annotated as rods, cones, BCs, ACs/HCs, RGCs, RPCs, MG, RPE cells and others. To further refine the annotation, we subsetted cells annotated as AC/HC, identified highly variable genes (default parameters) on the AC/HC subset, scaled the data, performed PCA (top 10 PCs), integrated data from different samples by MNN^69^ using the wrapper function in the R package SeuratWrappers with default parameters and performed Louvain clustering on the MNN representation (resolution = 0.5). Among those clusters, distinct ACs and HCs were identified. A similar procedure was applied to cells annotated as RGCs. Among the Louvain clusters (resolution = 0.3), cells were split into RGCs and precursors, and nine annotated cell types (RPCs and eight terminal cell types—rods, cones, BCs, ACs, HCs, RGCs, MG and RPE) were considered as well-defined cell types, whereas the rest of cells were considered as intermediate cell states. The average expression profiles of each cell type and the normalized transcriptomic similarity between cell types were calculated. Cell type annotation and normalized transcriptomic similarities were transferred to the scATAC-seq atlas using the network propagation procedure described above, based on a new weighted *k*-nearest neighbor graph from the recalculated LSI, CSS and Harmony representation of the cleaned-up cells in the scATAC-seq data.

Marker genes were identified by comparing expression between cell types using the presto package in R combining multiple criteria (Benjamini–Hochberg-corrected two-sided Wilcoxon test *P* < 0.01, area under the curve (AUC) > 0.7, average fold change (FC) > 1.2, detection rate difference >20%, being detected in fewer than 20% of the other cells and excluding mitochondrial and ribosomal genes), ordered by cluster detection rates. Marker accessible peaks were defined as 1 with Benjamini–Hochberg-corrected two-sided Wilcoxon test *P* < 0.01, AUC > 0.51 and the ratio of detection rates in/out of the cluster >5, ordered by the detection rate differences.

scVelo^70^ was performed on the scRNA-seq data based on the stochastic RNA velocity model. The PCA-reduced CSS representation was used as the transcriptomic data representation. The RNA velocity transition matrix and velocity pseudotime were both obtained with default parameters. CellRank analysis^33^ was then applied to the same cells using a hybrid kernel (50% velocity kernel and 50% velocity pseudotime kernel), with the eight terminal cell types considered as terminal states, to calculate the terminal state probabilities. Network propagation was used to transfer both the velocity pseudotime and the terminal state probabilities to other cells, based on the unweighted *k*-nearest neighbor(*k* = 50) graph calculated from the PCA-reduced CSS representation.

We constructed a graph abstraction of the differentiation trajectories to different cell types from RPC as follows. Cells were grouped into three groups—ACs, HCs and others—and Louvain clustering (resolution = 20) was applied to the PCA-reduced CSS representation of cells in each group to get high-resolution clusters, which were then pooled, as the cell state representatives. Cell metadata, including categorical (for example, cell type annotation) and numeric (for example, velocity pseudotime and CellRank terminal state probabilities) information, were summarized to the clusters with either max-pooling or mean. PAGA, as implemented in scanpy, was used to compute connectivity between clusters given the PCA-reduced CSS representation (n_neighbors = 20). Two clusters were connected if all the following three criteria were satisfied: PAGA connectivity >0.2; one cluster being one *k*-nearest neighbor (*k* = 20) of the other cluster in the summarized CellRank terminal state probability space; and the two clusters do not belong to different terminal cell types. The connection was directional, from the cluster with lower pseudotime to the one with higher. A force-directed layout of the graph was generated for visualization.

To further refine cell type branching and trajectory estimates, we chose five clusters with the lowest velocity pseudotime as roots and performed 100,000 random walks toward a node with zero to-degree, discarding non-terminal nodes. For random walks reaching each of the eight terminal cell types, the frequencies of passing by each node in the graph were counted and normalized by the number of random walks reaching that terminal cell type. For each node, the resulting normalized frequencies to different terminal cell types were further normalized by the sum to get the estimated likelihoods of the cell state differentiating into different terminal cell types. Any likelihood less than 1% was set to 0 and renormalized to get the final likelihood matrix.

### Reconstruction of GRN governing retinal organoid development

We applied Pando^34^ to the pseudocell-summarized data of the RNA-ATAC paired portion of the retinal organoid timecourse data to infer the GRN. Pando incorporates evolutionary conservation, prior regulatory elements, data-driven open accessible regions (for example, peaks called in the scATAC-seq data), TF motif database and binding site prediction to identify putative *cis*-regulatory elements and putative *trans*-regulators (that is, TFs) of each gene and fits a linear model of gene expression against interactions of the *cis*-regulatory element accessibility and *trans*-regulator expression, followed by statistical tests to define significant TF-motif-target triplets. Pseudocells were constructed by averaging transcriptomically similar cells from the same cluster of the same sample, following the procedure as described previously^71^ and implemented in the simspec package^30^, with selection ratio of 0.1. This procedure was to decrease the noise due to data sparseness, particularly chromatin accessibility data. The extended TF binding motif database was constructed using a similar procedure as described previously^34^, integrating JASPAR (2020 release)^72^, CIS-BP database^73^ and sequence-similarity-based motif transfer. Pando was run in default setting, except for the tf_cor threshold being 0.05. A TF-motif-target triplet was considered as significant if Benjamini–Hochberg-corrected *P*<0.01.

To generate the layout of the resulting GRN highlighting TFs, the pairwise Pearson correlations of gene expression between different genes across the cell state representatives defined above were first calculated as the base TF–gene linkage score. Next, the lineage score between any TF–gene pair with no inferred direct regulatory relationship was set to 0. PCA (top 20 PCs) was then applied to convert the linkage score matrix to represent each TF, which was used as the input to generate the UMAP embedding of the TFs. For each TF in the GRN, its expression pseudotime was calculated as the average pseudotime of cell state representatives weighted by the expression level of the TF in different cell state representatives. The pseudotime dependency of the TF is calculated as R^2^ between its expression across cell state representatives and the predicted values of a smooth splines model of expression against pseudotime (degree=3).

### Multimodal spatial integration

To integrate the spatially resolved time course of 4i nuclei with the multimodal single-cell sequencing (scSeq) dataset, we established an integration pipeline in Seurat (version 4.0.0) in which we perform high-resolution clustering in RNA space and transfer these metacluster labels to the 4i data by CCA anchoring. Protein stain intensity features of segmented 4i nuclei from the organoid samples of the developmental timecourse and the primary adult sample were log(*x* + 1)-transformed and imported into individual Seurat objects. Several protein stains were excluded (Supplementary Table 1) due to prior quality assessment and protein stains that do not have an associated gene in the RNA expression data (MAPK1/MAPK3, Serotonin, NPC and Hoechst). For each timepoint in the developmental timecourse (weeks 6, 12, 18, 24 and 39), the respective samples were integrated by CCA anchoring using the Seurat functions FindIntegrationAnchors(dims = 1:10) and IntegrateData(dims = 1:10). Subsequently, PCA was performed on the integrated samples, and UMAP embeddings were generated from the first 10 PCs for each timepoint. The adult sample was processed in the same manner but skipping the integration procedure.

Integration was performed for each timepoint of the 4i dataset separately by selecting respective matching and/or adjacent timepoints in the multimodal scSeq data. To maximize the proportion of ATAC-paired cells in the subsets, we matched week 6 of the 4i data with week 6 of the multimodal scSeq data; week 12 with weeks 11, 12 and 13; week 18 with week 18; week 24 with week 24; week 39 with weeks 36, 38 and 40; and the adult sample with weeks 36, 38, 40 and 46, respectively. For each subset of the multimodal scSeq data, Harmony integration^68^ was run accounting for sample grouping to evenly distribute paired ATAC cells among high-resolution metaclusters that were subsequently computed with the Seurat function FindNeighbors(resolution = 30, dim = 15). To account for low total numbers of paired ATAC cells in matched subsets for weeks 24 and 39, we imputed ATAC signals for cells with no ATAC information within respective *k*-nearest neighbor graphs by applying network diffusion (*n* = 20). RNA expression, ATAC peak regions and TF motif score matrices were averaged by assigned metaclusters of each respective timepoint. To spatially resolve RNA expression, ATAC peaks, cell-type-specific ATAC peak sets and TF motif scores, we transferred cell type and metacluster labels from matched multimodal scSeq subsets to the corresponding 4i nuclei data subsets by CCA anchoring. For visualization of the mapped ‘Intermediate’ cell types in UMAP embeddings and onto organoid images, we calculated mixed colors based on the maximal correlation of intermediate cells with the defined terminal cell states for the multimodal scSeq data and further averaged these by metaclusters for visualizing 4i nuclei cell types. We added the position on the inner–outer axis of each nucleus to the metadata by calculating the Euclidean distances to the respective organoid contour outlines. We also added the pseudotime rank of respective laminar windows as a binary count matrix that accounts for nuclei that might be positioned within overlapping neighboring laminar windows. For visualization of cell type and feature distributions along the inner–outer axis and pseudotime trajectory of laminar windows, the integrated multimodal datasets were filtered for nuclei that occur at least in one laminar window with assigned pseudotime rank and then plotted as density contour estimates from pseudotime and inner–outer axis resolved cell type frequency or amplified feature count matrices. Count matrices for cell type abundances were generated by aggregating discretized nuclei positions along the inner–outer axis. To map continuous features, such as RNA expression or ATAC peak regions, count matrices of all nuclei were multiplied by their respective scaled (0–1) feature matrix, amplified by a factor of 10, rounded and aggregated.

### EPHB2 spatial correlation analysis

Variable features (weeks 38 and 40) were detected with the Seurat function FindVariableFeatures(). We then calculated two-dimensional kernel density estimations with an axis-aligned bivariate normal kernel for all respective per nuclei resolved spatial features across all organoid sections of week 39 using the kde2d() function from the R package MASS^74^ on a 200 × 200 grid. In addition, we calculated respective binary mask grids by calculating density estimations just based on nuclei positions and applying a threshold of 0.05. We then calculated the respective spatial correlations of all selected features with EPHB2 across all organoid samples after multiplying respective density estimations with their corresponding binary mask. Pearson correlations were then averaged by feature across all analyzed organoid sections. In addition, we calculated the spatial correlation of cell type distributions with EPHB2 in the same manner as described above, selected the top 115 positive and negative correlated features and performed GO term enrichment using the R package clusterProfiler^75^ setting the background genes to the complete human genome. For visualization, we plotted heat maps of the top four positive and negative correlating features as well as EPHB2 in all analyzed organoid sections. Correlation of cell type distributions with spatial EPHB2 expression was visualized as a heat map after applying hierarchical clustering (Ward.D2) of cell types across all analyzed sections. Results of the GO analysis were visualized as dot plots where we denote the detected gene ratios and −log_10_(adjusted *P* values). A complete list of input genes and resulting GO terms is shown in Supplementary Table 4.

### Differentiation trajectory and cell neighborhood analysis of RGCs

To reconstruct RGC differentiation in week 6 retinal organoids from either RNA or protein information, averaged scaled RNA (for week 6 detected variable features) and protein abundances for all week 6 high-resolution metaclusters in the 4i data were calculated. Subsequently, RNA and protein metaclusters were assessed separately by diffusion analysis, and respective diffusion pseudotimes were obtained. For visualization, we calculated averaged mixed colors for each metacluster, averaged CellRank probabilities for RGCs by metacluster and applied hierarchical clustering (Ward.D2) to 25 RNA transcripts that showed the highest absolute Spearman correlation against pseudotime ranks and six transcripts whose corresponding 4i signals correlated with pseudotime (*HES1*, *SOX9*, *VSX2*, *PAX6*, *POU4F2* and *ONECUT2*).

To analyze local, microenvironmental variation of RGC cells in the 4i data, we performed spatial resolved neighborhood analysis of protein and MTU signals within week 12 retinal organoid sections. We segmented RGC neighborhoods through a 40-pixel (6.5-μm) radial extension from each nuclei centroid and averaged respective protein signals by radial position. We masked respective nuclei to exclude protein intensity signals that stem from the nuclei themselves and to focus on the respective radial neighborhoods. We then processed the retrieved radial intensity profiles and reconstructed laminar organization dynamics as described above. In brief, we first applied *z*-scoring and scaled the intensity profiles between 0 and 1, smoothed the signals by applying a 1D mean filter (window size = 20), downsampled by a factor of 2 and calculated respective distance matrices by applying fast Fourier transform and averaging the Euclidean distances of the first 10 frequency components. We then assessed the heterogeneity of RGC microenvironments by applying diffusion analysis, calculating UMAP embeddings from the first 10 DCs and clustering radial neighborhoods by performing Louvain clustering on the UMAP embedding. Assigned Louvain clusters were then mapped onto respective week 12 organoid sections for visualization. We further averaged the MTU abundances within each Louvain cluster’s radial neighborhoods and applied hierarchical clustering (Ward.D2) and visualized the results in a heat map. For further visualization of the detected radial spatial neighborhoods, we randomly selected six neighborhoods from all analyzed week 12 sections for each detected Louvain cluster and cropped respective image collages for Hoechst stain, all MTUs and an RGB overlay of POU4F2, HSPD1 and TUBB3 protein stains as well as a selection of MTUs 6, 8, 13, 17 and 20.

As we observed RGC nuclei with apoptotic morphologies in several Louvain cluster neighborhoods, we further searched for apoptosis-related processes in the scRNA-seq data of RGCs in weeks 11, 12 and 13. We, therefore, retrieved a list of human genes associated with the GO term apoptotic process (GO:0006915) from the AmiGO 2 database (http://amigo.geneontology.org/amigo/term/GO:0006915), which includes genes that are positively and negatively regulating apoptosis. We detected variable transcripts of RGCs by using the Seurat function FindVariableFeatures(). Next, we performed PCA for detected variable features that are also present in the retrieved list of apoptosis-related genes and used the calculated PCA to run Harmony integration accounting for sample groupings. Based on the first 30 dimensions of the Harmony embedding, we assigned clusters with the Seurat functions FindNeighbors() and FindClusters(resolution = 1) and calculated a UMAP embedding. Gene markers of each detected RGC cluster were identified by comparing expression of cells of one cluster to cells of other cluster cell types using the presto package in R for all detected variable features. Cluster markers were selected by combining Benjamini–Hochberg-corrected two-sided Wilcoxon test *P* < 0.05 and AUC > 0.5 criteria. We selected the top 50 markers from this ranking and used DAVID to perform GO term enrichment analysis and KEGG pathway analysis for cluster 6, which was defined by a set of marker genes strongly relating to apoptotic processes (Supplementary Table 5). We calculated a score for this detected apoptotic signature by using the Seurat Function AddModuleScore(ctrl = 5) for the top 10 markers of cluster 6. For visualization, we created feature plots of the UMAP embedding for the detected clusters as well as *POU4F1*, *POU4F2*, *DDIT3*, *SCG2*, *ATF3* and apoptotic module scores of cluster 6.

### Processing and multimodal integration of multiplexed smFISH data

Spot segmentation and pre-processing of smFISH image data were performed as described^76^. To assign the detected spots of the multiplexed smFISH data to individual cells, we applied Baysor^77^ for cell segmentation. As a prior input for segmentation, Cellpose-labeled nuclei masks from all 20 organoid slices were generated (model = ‘cyto2’, diameter = 50, flow_threshold = −3, cellprob_threshold = 0.8). Baysor was run with default settings and a specified initial estimated cell radius of 25 to obtain respective expression matrices, spot cell assignments, centroids and convex hulls of the segmented nuclei. For the visualization of spatial transcript distribution, we generated spatial multi-transcript plots as well as faceted insets of respective laminar organized tissue for a selection of high abundant transcript of both timepoints. We colored all measured transcripts in the same insets by assigned cell and generated an overlay of DAPI images (week 13 and week 32) and their respectively colored convex hulls for representative laminar organized tissue areas.

For integration of the spatially resolved FISH nuclei with the multimodal scSeq dataset, we used an integration pipeline similar to the 4i integration pipeline. In brief, we imported Baysor expression matrices into Seurat objects, and, for each timepoint in the FISH dataset (weeks 13 and 18), the respective samples were integrated by CCA anchoring using the Seurat functions FindIntegrationAnchors(dims = 1:10) and IntegrateData(dims = 1:10). Then, PCA was performed on the integrated samples, and UMAP embeddings were generated from the first 10 PCs for each timepoint. Cell type label and high-resolution metacluster transfer from the multimodal scSeq datasets to the spatially resolved FISH nuclei were performed in the same manner as described above for the 4i nuclei. For integration, we matched weeks 11, 12, 13 and 15 to the week 13 smFISH samples and weeks 30, 31, 32 and 34 to the week 32 smFISH samples. For visualization purposes, we resolved labeled ‘Intermediate’ cell types in the shown UMAP embeddings and organoid overlays with the same color mixing approach as for the 4i nuclei. Furthermore, we averaged transcript abundance by assigned cell type labels, *z*-scored average expression across transcripts, applied hierarchical clustering (Ward.D2) and visualized the results as a heat map.

### Multimodal spatial comparison of 4i and smFISH

To generate laminar windows for the smFISH data, Laminator analysis was applied in a similar manner as for the 4i data. Initial masks for each organoid sample were created by generating binary images of all located spots per sample and applying a combination of dilation, fill holes and erosion binary operations, followed by Gaussian blurring, Otsu thresholding and selection of the largest segmented area (for details, see GitRepo). Tiles that were not imaged were further masked by thresholding corresponding DAPI images and combining the tile masks with their corresponding initial masks. Similarly to the above-described Laminator analysis, organoid contours were detected, and laminar windows (100 × 1,000 pixels) were placed and oriented at the same intervals along the contours. We generated individual binary images for all transcripts from the respective discretized spatial coordinates, extracted intensity profiles and applied filtering for straight contour areas that stem from unimaged tiles, reverse *z*-scoring, smoothing and downsampling of retrieved intensity profiles, distance matrix construction and averaging, diffusion analysis and Louvain clustering of laminar windows with the same parameters as for the 4i data.

We compared and matched smFISH laminar windows with the spatiotemporal laminar window trajectory of the 4i dataset. Segmented smFISH and 4i nuclei were assigned to their respective laminar windows, and their position on the inner–outer axis was determined. Laminar windows (smFISH and 4i) were separated into equally spaced zones (*n* = 6) along the inner–outer axis, and pseudobulk transcriptomes were calculated from the respective assigned nuclei’s scaled expression values. Note that, for the 4i nuclei, we used the predicted scaled expression values of the transcripts overlapping with the measured transcripts of the smFISH data. Averaged Euclidean genomic distances of matched spatial zones between all smFISH and 4i windows were calculated. Consequently, each smFISH window was paired with the 4i window with the respective minimal averaged genomic distance if the same spatial zones were populated by nuclei as in the compared smFISH window. To visualize the similarity of smFISH to 4i windows across experiments, we grouped matched window pairs by 4i and smFISH timepoint and plotted the respective distributions of averaged genomic distances as box plots.

To assess spatially constrained correlation between measured and predicted RNA abundance, we further matched nuclei across matched smFISH and 4i laminar windows. Each nucleus in the smFISH windows was paired with the 4i nucleus that had maximal Pearson correlation between the smFISH scaled expression of transcripts and predicted scaled transcript expression in the 4i nuclei. From these paired multimodal metacells, which possess protein-level, predicted RNA levels from 4i nuclei as well as measured and predicted RNA levels from smFISH, intermodality correlations for each of the measured transcripts of the smFISH data were calculated for week 13 and week 32 smFISH samples. For visualization of predicted and measured RNA levels and protein levels, we calculated the respective averaged inner–outer intensity profiles for all modality types of *VSX2*, weighting 4i laminar windows that were matched multiple times with log(*n* + 1). In addition, we added the respective 1–99% confidence intervals for the null hypothesis of Pearson correlation and log_10_(*n* + 1) counts of total transcript abundances to related bar plots and scatter plots.

### Power analysis 4i and smFISH

To estimate the power of distinguishing different cell types or subtypes using the 4i and smFISH data, we curated a graph of clusters with varied resolutions on transcriptome in a hierarchical manner, based on the scRNA-seq data. The classification of cells into eight major cell types (RPCs, RGCs, HCs, ACs, BCs, cones, rods and RPE) and the intermediate state, which covers all the other non-initial and non-terminal cell states, is considered as the first layer (L1). The second layer of clustering (L2) expanded the intermediate state into nine different clusters, the RPC cluster into G2M-phase RPC and other RPC clusters and the BC cluster into BC-on and BC-off clusters, all based on the Louvain clustering (resolution = 0.3) mentioned above. Afterwards, cells of different L2 clusters were split, and the Louvain clustering was applied to each subgroup with varied resolutions (0.1, 0.5 and 1) to obtain subtypes of each cell type, which altogether defined the third to fifth layer of clustering (L3–L5). Finally, the cell states used for the above-mentioned graph abstraction of the differentiation trajectories were considered as the finest layer of clusters (L6). The average transcriptome profiles were calculated for every cluster on different layers by averaging the transcriptome profiles of cells assigned to the cluster.

To visualize the hierarchy of clusters, we represented different layers of clusters as a graph, where each cluster was a node, and two clusters were connected by an edge only if they were from two nearby layers and the two clusters had at least one shared cell. The edge was weighted as *n*_*ij*_ / *n*_*i*_, where *n*_*ij*_ is the number of cells shared by the two clusters, and *n*_*i*_ is the number of cells in the cluster at the coarser layer. Edges with weights less than 0.1 were trimmed. The cluster graph was then visualized using the ggraph package in R with the tree layout implemented in the igraph package (the layout_as_tree function), with the edge weights represented by the edge alpha-transparency.

Next, the Pearson correlation coefficient (PCC) was calculated between the average transcriptome profiles of each cluster and the protein abundance profile of every segmented nucleus in the 4i experiments. For each 4i nucleus, PCC was calculated for all L1 clusters, whereas, for the other layers, only clusters reachable from the L1 cluster that the nucleus was predicted to be in the integration procedure mentioned above were included. The power of discriminating different clusters at different layers for the 4i experiment was represented by the distribution of differences between the maximal and the second-largest PCC in all the 4i nuclei. A similar procedure was also applied to the Baysor segmented cells in the smFISH experiments. To estimate the background of the discrimination power, cells of each L1 cluster were randomly grouped into two groups, each of which with the average transcriptome profiles calculated. For each 4i nucleus or smFISH segmented cell, PCCs were calculated to the two average transcriptome profiles of the cluster it was predicted to be. The distribution of differences between the two PCCs across all 4i nucleus or smFISH cells was considered as the background of zero discrimination capacity.

### Vector and lentivirus preparation for perturbation experiment

The lentiviruses for perturbation experiment were produced as described^34,40^ with minor modifications. In brief, a modified CROP-seq vector carrying GFP^34^ was used. Three gRNAs per targeted gene (*NRL*, *OTX2*, *PAX6*, *VSX2* and *CRX*) were designed using the GPP Web Portal (https://portals.broadinstitute.org/gpp/public/) and synthesized by IDT following^40^ recommendations. Moreover, a non-targeting ‘dummy’ guide (CGCTTCCGCGGCCCGTTCAA) was added. Oligonucleotides were pooled in equal amounts and assembled in the vector backbone by Gibson isothermal assembly. The plasmid library was sequenced to validate the complexity of the pooled plasmid library, and 10 ng of plasmid library was used for generating a sequencing library with a single PCR reaction. Illumina i7 and i5 indices were added by PCR, and the library was sequenced on Illumina’s MiSeq platform. Upon validation, lentiviruses were generated.

### ‘In organoid’ CROP-seq perturbation experiment

Retinal organoids derived from 409B2-iCas9 line at 19 weeks of development were infected with the lentiviral pool described above. Twenty organoids were individually infected in ultra-low attachment 96-well plates (Costar). Each organoid was infected with 1 µl of LV (titer = 5.61 × 10^8^ TU ml^−1^) in 100 µl of medium. After 4 h, 150 µl of medium was added for O/N incubation. The day after, organoids were moved to 10-cm plates and treated with doxycycline 4 µg ml^−1^ for 1 week to induce Cas9. After 3 weeks, 10 organoids were dissociated with the papain-based dissociation kit (Miltenyi Biotec, 130-092-628) described previously, and GFP^+^ cells were enriched by fluorescence-activated cell sorting (FACS) (Experiment 1). The other 10 organoids were processed 5 weeks after infection (Experiment 2). We obtained a total of 12,000 and 7,000 GFP^+^ cells, respectively, and loaded them for scRNA-seq. We note that the first sample experienced a potential wetting error during GEM generation. scRNA-seq libraries were generated with the Chromium Single Cell 3′ version 3.1 Library & Gel Bead Kit. The expression libraries were FAB treated and sequenced on Illumina’s NovaSeq platform.

### gRNA detection from single-cell cDNA

gRNA sequences from single cells were amplified from 30 ng of scRNA-seq cDNA as described in ref. ^34^. The first PCR amplifies a broad region targeting the outer part of the U6 promoter. The second, nested, PCR targets the inner portion of the U6 promoter adjacent to the guide sequence and adds a TruSeq Illumina i5 adapter. Finally, a third PCR adds Illumina sequencing i7 adaptors. PCRs were monitored by quantitative PCR to avoid over-amplification. The samples were purified using SPRI beads (Beckman Coulter), and libraries were sequenced at 1:10 proportion of the transcriptome library on Illumina’s NovaSeq.

### CROP-seq data pre-processing and analysis

CROP-seq experiment reads were aligned to the human reference (GRCh38, 10x Genomics, version 3.1.0) with Cell Ranger (10x Genomics, version 4.0.0) to generate the transcript count matrix for each sample. The amplicon sequencing reads detecting the gRNAs were aligned to the extended human reference (GRCh38-based, 10x Genomics, version 3.1.0) using Cell Ranger (version 4.0.0) adding a guide-GFP construct as an artificial chromosome. A quality control procedure^34^ was used to extract informative gRNA transcripts detected in each cell based on a Gaussian mixture model of number of reads per unique molecular identifier (UMI), resulting in a guide transcript count matrix for each sample, which was binarized to obtain the final cell-to-gRNA and cell-to-target assignments.

CROP-seq transcriptomic data were merged and normalized using Seurat (version 4.0.0). Highly variable genes were identified (vst method, 3,000 genes, excluding cell-cycle-related genes and mitochondrial and ribosomal genes), and data scaling and PCA (top 20 PCs) were then performed, followed by data integration by CSS and further PCA reduction. The data were projected to the CSS space^30^ of the retinal organoid timecourse, and the *k*-nearest neighbor (*k* = 50) of each cell (*i*) in the CROP-seq data in the timecourse cell atlas (denoted as *N*^*r*^_*i*_), as well as the average of their distances in the CSS space (denoted as *d*_*i*_), were obtained. The average distance of each cell (*j*) in the timecourse atlas to its *k*-nearest neighbor (*k* = 50) within the atlas (*d*′_*j*_) was also calculated. A normalized projected distance of each cell in the CROP-seq data to the timecourse atlas was, thus, defined as

$$\underline {\mathbf{d}_i} = {\mathbf{d}}_i/\left( {\mathop {\sum}\limits_{j \in N_i^r} {\mathbf{d}}^\prime_j/|N_i^r|} \right).$$

The bimodal distribution of ${\underline {d_i}}$ suggested a group of cells with large projection distance to the reference data, which implied failure of the projection—that is, cell types/states that did not exist in the reference data. A Gaussian mixture model was then fitted to identify those cells that were excluded from the following analysis. A UMAP embedding was generated, and Louvain clustering with varied resolutions (0.2 and 0.6) was performed, both based on the PCA-reduced CSS representation of the remaining cells.

To estimate the perturbation probability of each cell being induced by the gRNA and Cas9 expression, we adapted the perturbation probability calculation^34^, using experiments and the Louvain cluster labels (resolution = 0.6) as covariates. The resulting perturbation probabilities were considered as the proxy of perturbation status of a cell and used in the following differential expression (DE) analysis. The DE analysis was applied to co-expression gene modules, which were defined in the retinal organoid timecourse cell atlas as follows. A *k*-nearest neighbor (*k* = 50) graph of genes detected in at least 1% of cells in the timecourse cell atlas was based on the Pearson correlation distance between gene expression across the abstracted cell state representatives. Louvain clustering (resolution = 10) was applied to the gene *k*-nearest neighbor graph to identify the co-expression gene modules. The gene module activities were quantified for cells in the timecourse atlas as well as cells in the CROP-seq data, separately, using the AddModuleScores function in Seurat with default parameters. An ANOVA-based DE method was then applied to the gene module activity scores of each co-expression gene module, testing whether the cell perturbation status of different targeting TFs significantly explained variance of the activity score of a gene module in the dataset, with additional covariates, such as experiments and cell clusters (resolution = 0.6), included in the model. Co-expression gene modules with Benjamini–Hochberg-corrected *P* < 0.1 were considered as differentially expressed modules (DEMs) caused by the perturbation of the targeting TF. The size of the DE effect was represented by a signed −log_10_(*P*), where *P* is the ANOVA *P* value and the sign being the sign of the estimated coefficient. The resulting DEMs were clustered using hierarchical clustering (Ward.D2 method), given the pairwise Pearson correlation distance across the timecourse cell state representatives. DAVID was used to do functional enrichment analysis of genes in each cluster of DEMs. The DE analysis was also applied to the two CROP-seq experiments separately with only the cluster label as the additional covariate, to estimate the robustness of the detected DE of the identified DEMs.

### Statistics and reproducibility

Imaging analysis and integration were performed on the entire datasets of the 4i and smFISH experiments, respectively. 4i and smFISH experiments were performed once each. They included multiple controls and analysis illustrating reproducibility and robustness of the procedures. All images and spatial plots presented are representative of the entire respective datasets or specific timepoints within the respective dataset if indicated. The 4i dataset included 16 organoids and one primary tissue. For each organoid timepoint, at least two organoids with at least two sections are represented with a total of 40 sections. The primary tissue sample is represented with a single section. See Supplementary Table 1 for details on the experimental design. For the smFISH data, four organoids per timepoint week 13 and week 32 were used. Week 32 organoids are represented with 12 sections and week 13 organoids with eight sections (Supplementary Table 7). All the box plots presented in figures were created using the default box plot setup in R. In brief, the midlines in boxes represent medians; the widths of boxes represent the lower and upper quartiles; the upper and lower whiskers represent values outside the middle 50% that are no more than 1.5 times the interquartile range from the boxes; and values out of the ranges are considered as outliers, which are shown as dots or not shown.

### Reporting summary

Further information on research design is available in the Nature Portfolio Reporting Summary linked to this article.

## Online content

Any methods, additional references, Nature Portfolio reporting summaries, source data, extended data, supplementary information, acknowledgements, peer review information; details of author contributions and competing interests; and statements of data and code availability are available at 10.1038/s41587-023-01747-2.

### Supplementary information

Supplementary Information Supplementary Figs. 1–15.

Reporting Summary

Supplementary Table 1 Overview of 4i experiments.

Supplementary Table 2 Overview of single-cell genomic experiments.

Supplementary Table 3 Cell type marker genes, accessible chromatin regions and Pando-inferred GRN.

Supplementary Table 4 EPHB2 spatially correlated genes GO term enrichment analysis.

Supplementary Table 5 Apoptotic RGCs GO term enrichment analysis.

Supplementary Table 6 SimpleElastix parameters.

Supplementary Table 7 Multiplexed smFISH supplementary information.

## Data Availability

All the data can be visualized and explored via the EyeSee4is app (https://eyesee4is.ethz.ch/). Compressed images in JPEG format, an example 4i image dataset in the raw TIFF format, multiplexed smFISH spot data, processed datasets of integrated scRNA-ATAC, 4i nuclei and smFISH nuclei as well as the 4i-scRNA-ATAC and smFISH-scRNA-ATAC integrated multimodal data are available via Zenodo (10.5281/zenodo.7561908)^78^. The raw sequencing data are available via ArrayExpress under accession number E-MTAB-12622 (CROP-seq)^79^. The scRNA-seq and scATAC-seq data are available at ArrayExpress under accession number E-MTAB-12714. The B7 cell line data are available in the European Genome-phenome Archive under accession number EGAS00001007065. The complete 4i image data of raw TIFF files are too large to provide on public repositories. They are available upon reasonable request to the corresponding authors.

## Code availability

Laminator and other code used in the analyses are available on GitHub (https://github.com/quadbiolab/spatial_multimodal_retinal_organoid).
