# Supplementary material for: Multimodal spatiotemporal phenotyping of human retinal organoid development
Source: Nat Biotechnol. 2023 May 8;41(12):1765–75. doi: 10.1038/s41587-023-01747-2 (PMC10713453; doi:10.1038/s41587-023-01747-2)
Supplement: Supplementary file 1 — Supplementary Figs. 1–15. [file 41587_2023_1747_MOESM1_ESM.pdf]

nature biotechnology

Article

https://doi.org/10.1038/s41587-023-01747-2

Multimodal spatiotemporal phenotyping of human retinal organoid development

In the format provided by the authors and unedited

Supplemental Figures

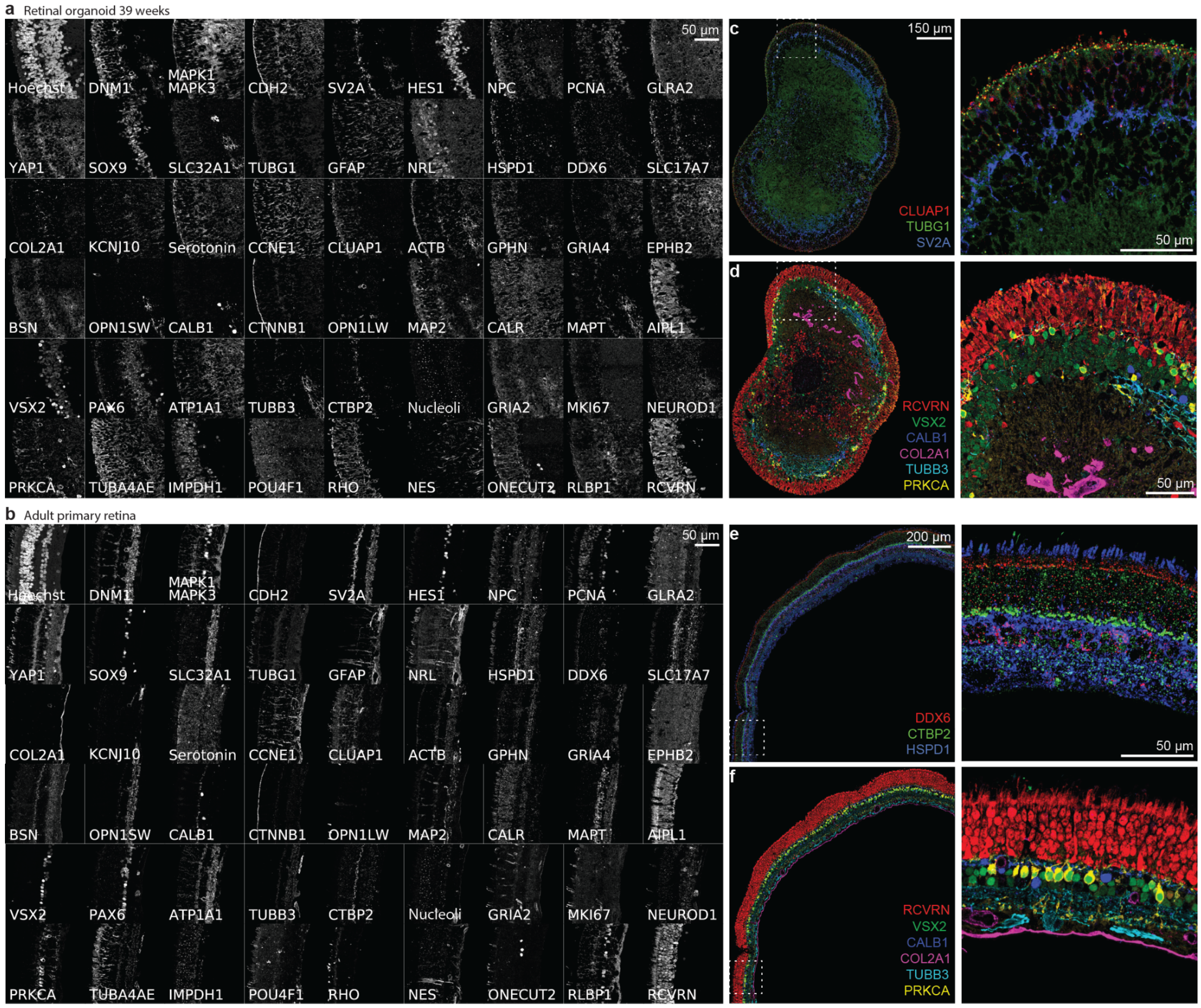

Supplemental Fig. 1: Representative images from primary adult human retina and a developed retinal organoid. a) Representative image segments from immunohistochemistry for each of the 53 antibody stainings and Hoechst over 21 cycles from a human retinal organoid at 39 weeks (a) or primary primary retina (b). All images are from the same representative region of the organoid or the primary retina respectively. All images were scaled to the top and bottom 1st percentile except TUBG1 which was scaled to the top and bottom 0.1 percentile. c-f) Representative three (c, e) or six (d, f) way overlays of a whole 39 weeks organoid section (c, d) or a primary retina tissue (e, f). c) TUBG1 and CLUAP1 highlight centrosomes and ciliary bases or tips respectively. SV2A marks synaptic layers. d,f) RCVRN marks photoreceptors, VSX2 and PRKCA bipolar cell nuclei or rod bipolar cells respectively, CALB1 marks horizontal cells, COL2A1 marks the inner limiting membrane in the adult primary sample (f) or undefined collagen rich structures in the organoid section (d). TUBB3 marks neurons. e) DDX6 marks P-bodies, CTBP2 ribbon synapses and HSPD1 mitochondria.

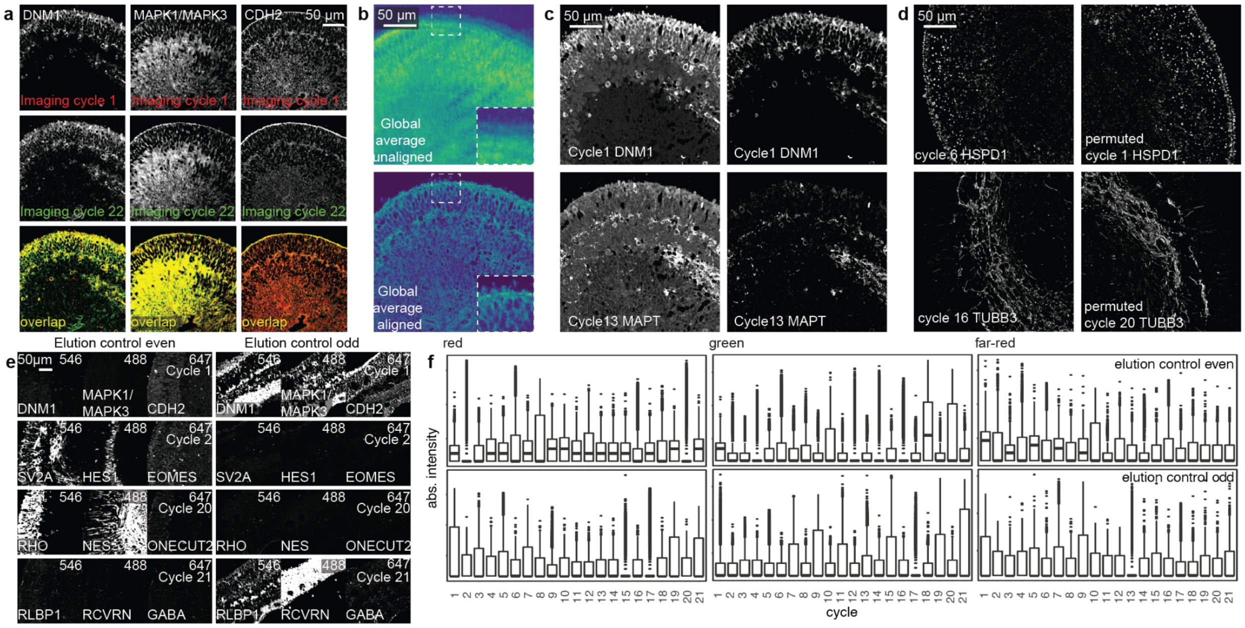

Supplemental Fig. 2: Quality control analysis of 4i data on tissues of developed retinal organoids. a) Images show the overlap (yellow) for three antibodies that were used for staining in cycle 1 (top row, red) and cycle 22 (bottom row, green) to assess restaining quality. b) Global average intensity signal of all stains (63 antibodies + Hoechst) before alignment (top), and after alignment (bottom). c) Images of representative stains, cycle 1 DNM1 (channel 546) and cycle 13 MAPT (channel 647) before (left) and after (right) background subtraction. For representation, each image was scaled to the top and bottom 2nd percentile of the signal intensity. d) Effects of the antibody order were tested by permuting the order in half the samples such that each organoid has sections stained in two orders. Shown are two example AB stainings (HSPD1, top; TUBB3, bottom) in original (left; HSPD1 cycle 6; TUBB3 cycle 13) or permuted order (right; HSPD1 cycle1; TUBB3 cycle20). e) Examples of staining, elution, and re-staining efficiency. Two kinds of elution controls were included in the experiment. On the left control “even” was stained in even numbered cycles and “eluted only” in odd numbered cycles and on the right control “odd” was stained in odd numbered cycles and “eluted only” in even numbered cycles. In both cases the three immunohistochemical channels are represented (546 left, 488 middle, 647 right). All images were scaled to the same absolute top and bottom value. Efficient elution was achieved until cycle 21. f) Signal changes over staining and elution cycles in elution control samples. The values were derived from fully processed (aligned, masked, denoised and background subtracted) images before exclusion of markers from the analysis. Masked images were randomly downsampled by a factor 1000 to select tissue pixels represented in the plots.

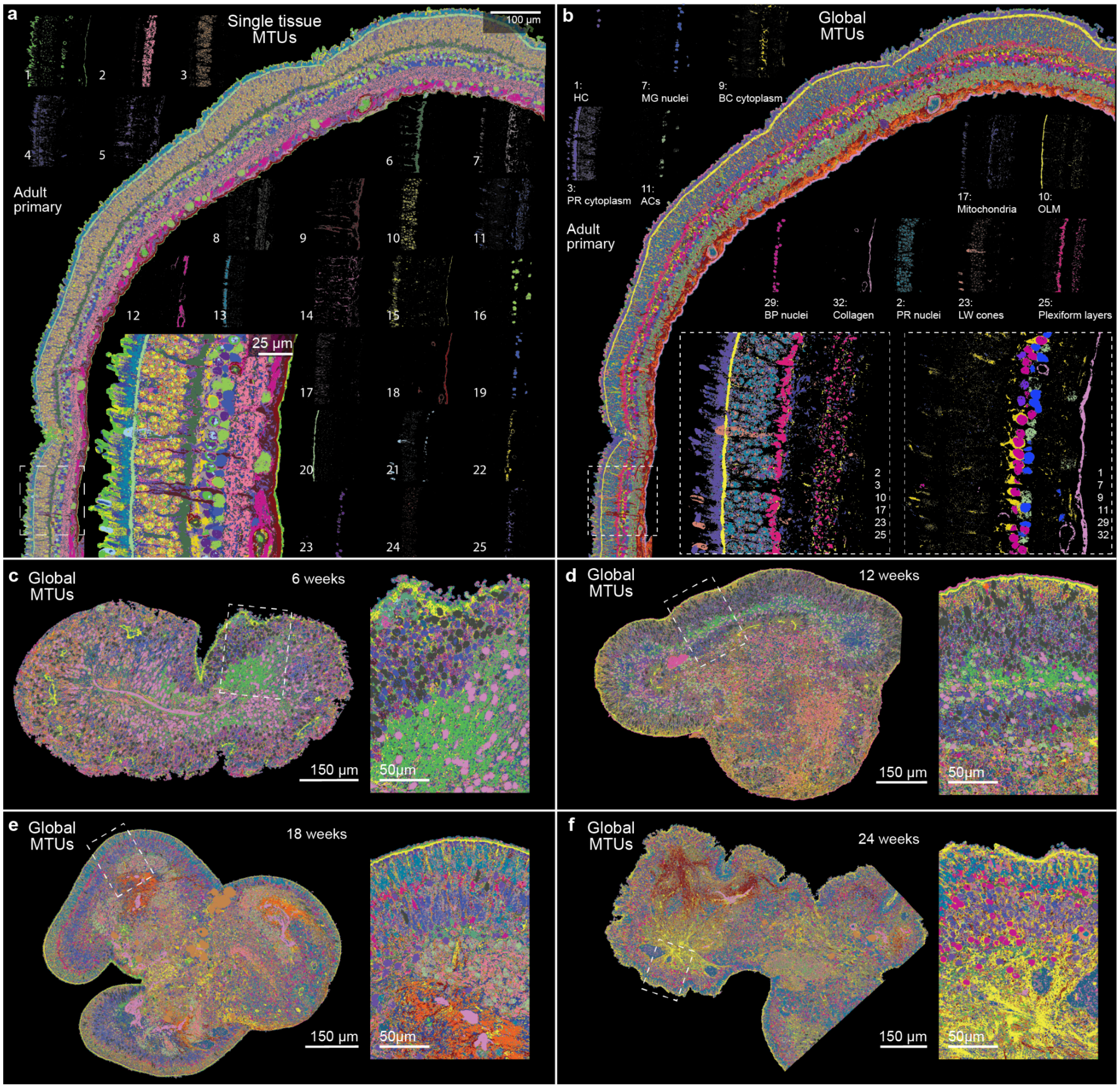

Supplemental Fig. 3: Assessment of single-sample MTUs in the adult retina and global MTUs across the organoid time course. a-b) Primary adult human retina section with pixels labeled by MTUs as generated by single sample clustering (a) or by global clustering including the entire organoid time course (b). Pixels assigned to each cluster are depicted individually for many of the clusters in each case. Highlighted clusters in b are the same as in Fig. 1e, f. Inset shows magnification of highlighted region (dotted line). Note that most MTUs have counterparts in single sample and global analyses. c-f) Pixel assigned MTUs in organoid sections over the time course**.** MTUs were derived from global clustering and are comparable across samples including the 39 week samples shown in Supplementary Figure 3.

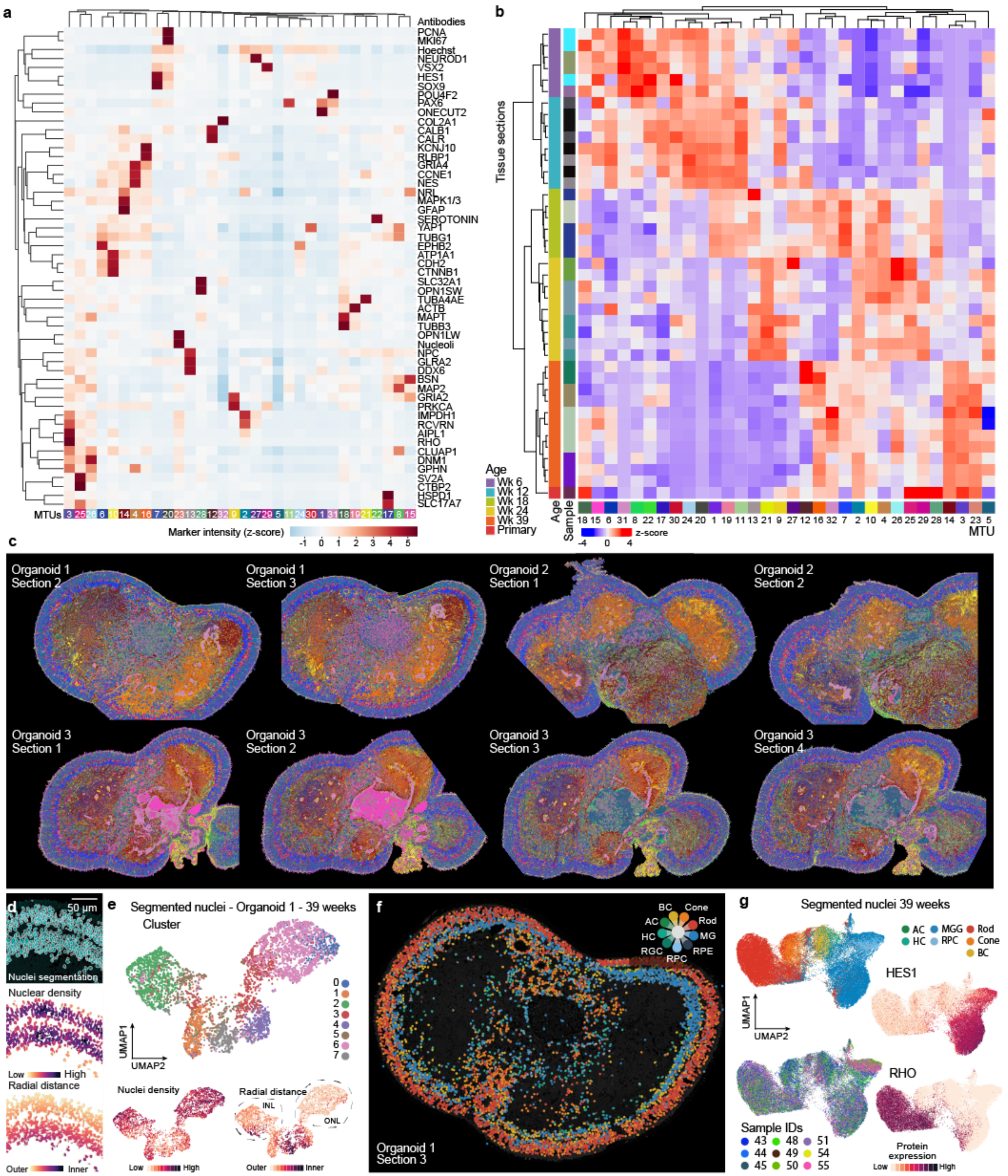

Supplemental Fig. 4: Extended analysis of 4i data on developed retinal organoids (39 weeks). a) Heatmap showing averaged protein detection intensity within each MTU. b) Heatmap of relative fractions of pixels assigned to each MTU per sample (z-scored, rows). Clustering highlights differences between time points, and consistency of MTU labeling within timepoints and between samples from the same organoid. c) Representative MTU images from three different 39 week organoids (6 out of 11 total sections analyzed from these organoids)

show qualitatively congruent label assignment at a global scale. d) Nuclei segmentation allows measurement of features per nucleus. Features measured include spatial features such as nuclei density, radial distance and others or protein expression levels and MTU abundance measures. e) UMAP embedding of median immunohistochemical signal detected per nucleus of a single 39 week organoid section. Color coding based on agglomerative clustering resulted in 8 clusters (top panel) that broadly agree with cell types transferred from scRNA-seq data. Color coding of Nuclei density and Radial distance (bottom panels) highlight increased nuclei density in the INL and ONL and radial localization of the respective nuclei. f) Cell types transferred from scRNA-seq data mapped back into a representative 39 week organoid section. g) UMAP embedding based on protein features of all nuclei of all 39 week organoid samples. Color coding based on cell types transferred from scRNA-seq data classifies nuclei into each of the major cell types. Signal intensity of HES1, RHO, and PRKCA highlight nuclei of the INL, ONL, and BC respectively.

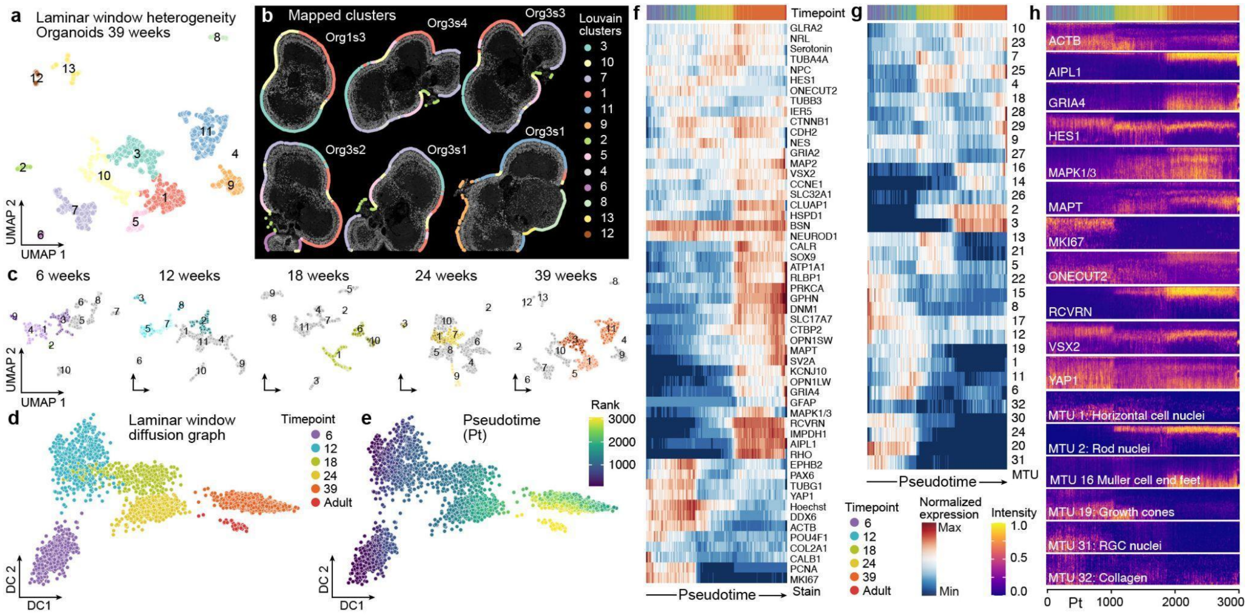

Supplemental Fig. 5. Extended analysis of laminar trajectory reconstruction. a) UMAP of laminar windows from all 39 week organoid 4i data colored and numbered by cluster. b) Image of each 39 week organoid section with window locations annotated as circles around the periphery and colored by cluster. Note the small disorganized regions in Org3s1, cluster 8. c) UMAPs from the window heterogeneity analysis for each time point, with the clusters colored that were used for trajectory reconstruction. d-e) Windows projected onto diffusion component 1 and 2 from multidimensional diffusion analysis colored by time point (d) or by pseudotime rank (e). f-g) Heatmaps showing the average expression of protein stains (f) or MTU abundances (g) across windows ordered by pseudotime. Top sidebar colors each window by time point. h) Heatmaps showing protein expression or MTU intensities across laminar windows ordered by pseudotime, where each x position is a window with radial intensities averaged and then scaled across the inner-outer axis.

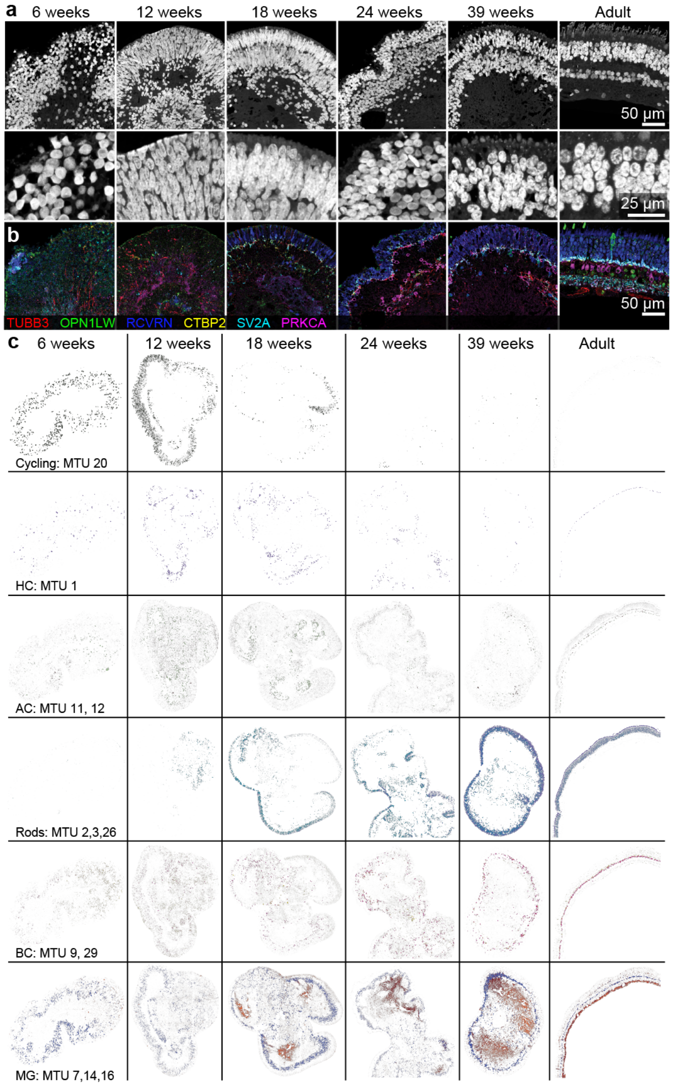

Supplemental Fig. 6. Analysis of features that change across the developmental time course. a) Hoechst stain in representative areas of organoids across the time course. Note the elongated nuclei at the periphery of the organoids at age 12 weeks and 18 weeks. b) Six color overlays in representative areas of organoids across the time course show emergence of synapses interfacing between neurons, BCs and PRs. TUBB3 (red) general neuron marker, OPN1LW (green) long wave cones, RCVRN (blue) photoreceptors, CTBP2 (yellow) ribbon synapses, SV2A (cyan) general synapse marker, PRKCA (magenta) rod bipolar cells. c) MTUs marking different cell states and types shown on organoid sections across the time course.

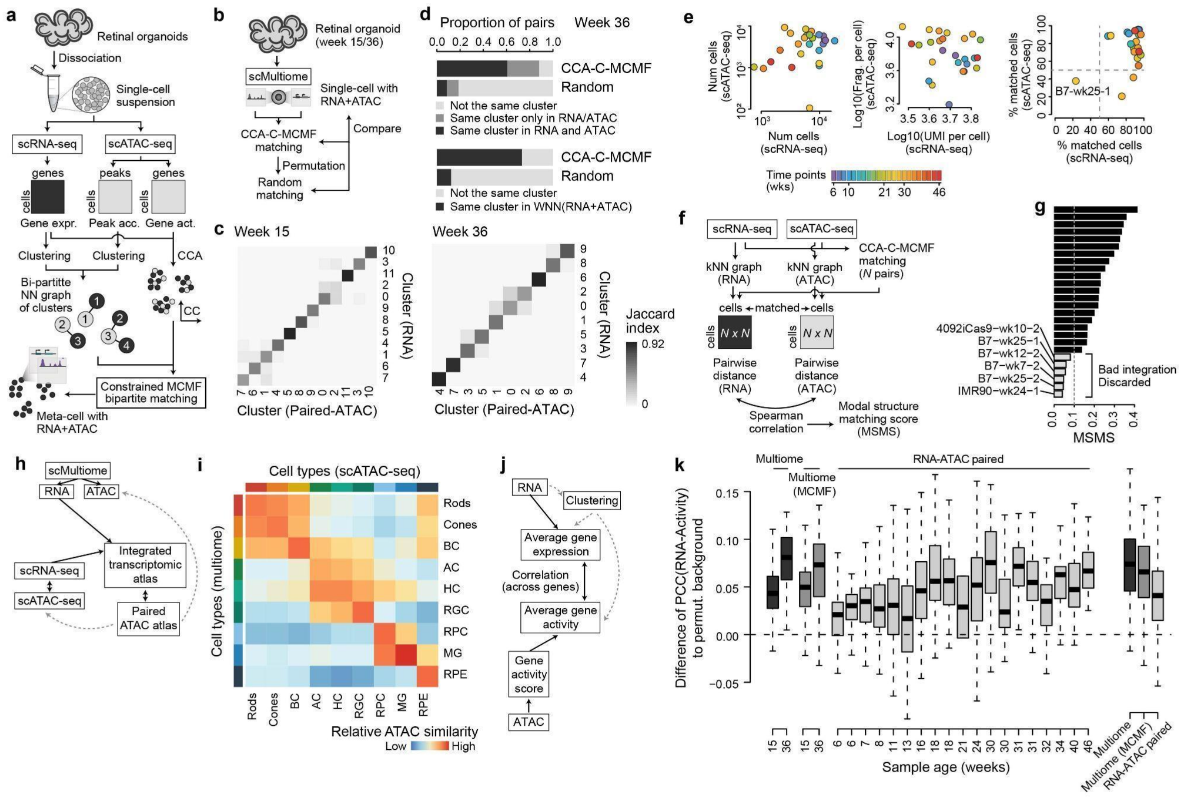

Supplemental Fig. 7: Supplemental analysis of single-cell multiomic integration. a) Schematic of the experimental design and data integration strategy for the scRNAseq and scATACseq developmental time course. Organoids for each time point were dissociated and the cell suspension was used for scRNA-seq and scATAC-seq (see Table S2 for details). Modalities were integrated using minimum-cost maximum-flow (MCMF) bipartite matching in CCA space. The resulting metacells with RNA and ATAC components are integrated using CSS. b) Schematic outlining the integration strategy for multiome sequencing data where mRNA and chromatin access were measured in each single cell. c) Pairwise Jaccard indices between clusters of cells and clusters of the paired cells using our constrained CCA-MCMF integration in multiome samples. d) Stacked barplots showing metrics assessing the metacell construction. e) Quality control assessments for the scATACseq datasets colored by time point. f) Schematic showing how multiome data was used to assess integration. g) Model structure matching score (MSMS) assessment of integration and rationale for sample discard. h-i) Schematic showing generation of the scSeq integrated multi-ome atlas, and heatmap showing the correspondence between cell types annotated based on scATACseq or multiome (RNA + ATAC, same cell) (i). j) Schematic showing the procedure to benchmark similarities between the RNA component and ATAC-derived gene activity score profiles. k) Boxplot showing distributions of RNA-ATAC similarities of genes at different samples, relative to the random distributions obtained by cell state label permutations. Colors of boxes show data types: dark grey - multiome, grey - multiome integrated with CCA-MCMF, light grey - RNA-ATAC paired samples integrated with CCA-MCMF

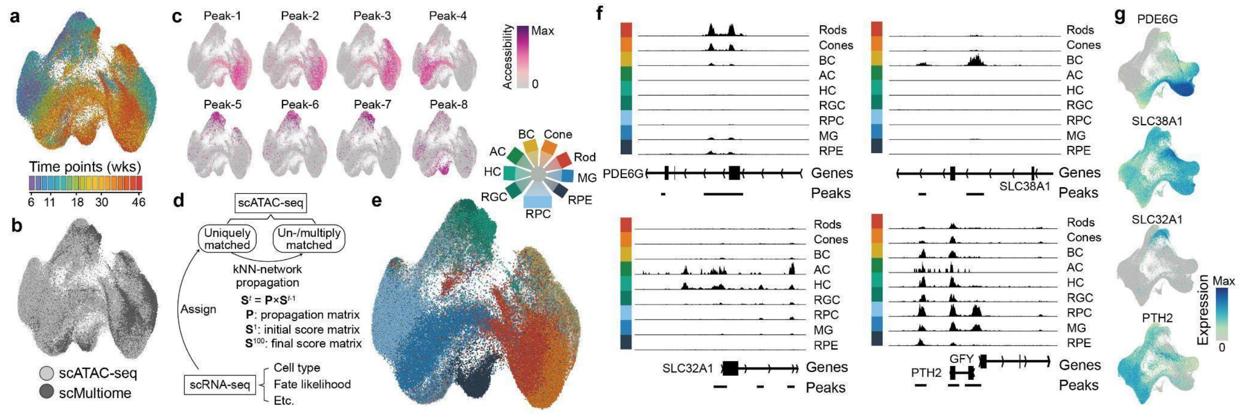

Supplemental Fig. 8 scATAC-seq data illuminates cell-type specific gene regulatory regions. a) UMAP embedding of scATACseq integrated with CSS and colored by time point (a), sample type (b), or by accessibility of cell type markers (c). d) Schematic showing the strategy for assigning labels to scATACseq cell types based on matching to cell types annotated from scRNAseq data. e) UMAP colored based on label matching from the scRNAseq data. f) Example of loci showing cell type-specific differential accessibility during retinal organoid development. Signal tracks show averaged chromatin accessibility in the different cell types. g) Transcriptome-based metacell UMAP colored based on genes shown in panel f.

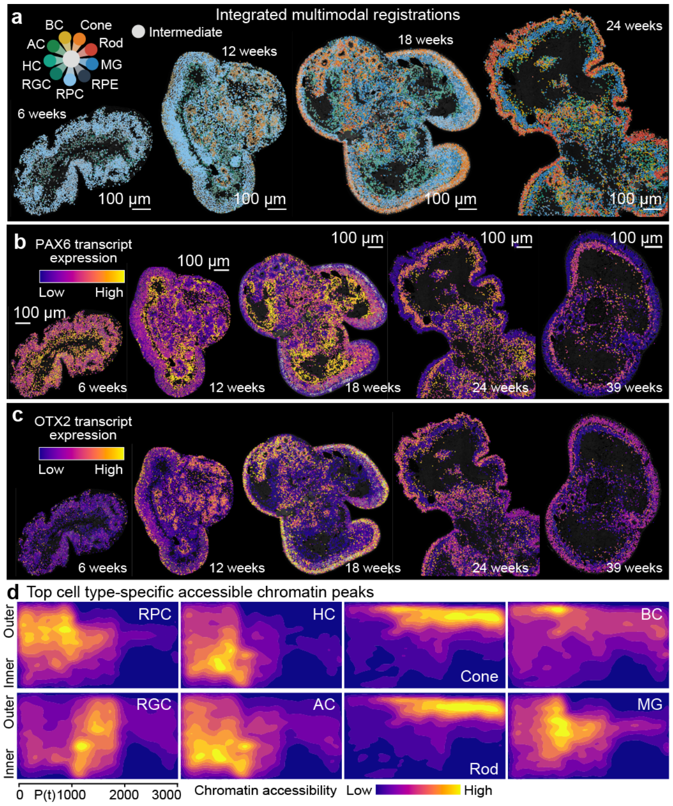

Supplemental Fig. 9: Integrated multimodal registrations with expression and chromatin access features across the retinal organoid time course. a) Overview of reference 6, 12, 18, and 24 week retinal organoid sections colored based on nuclei-type assignment from the label transfer. A corresponding 39 week organoid section is in Fig. 4b. b-c) Reference 6-39 weeks retinal organoid nuclei colored based on PAX6 (b) or OTX2 (c) RNA expression. d) Heatmap showing average chromatin accessibility densities of cell type-specific peaks for the major annotated retinal organoid cell types/states.

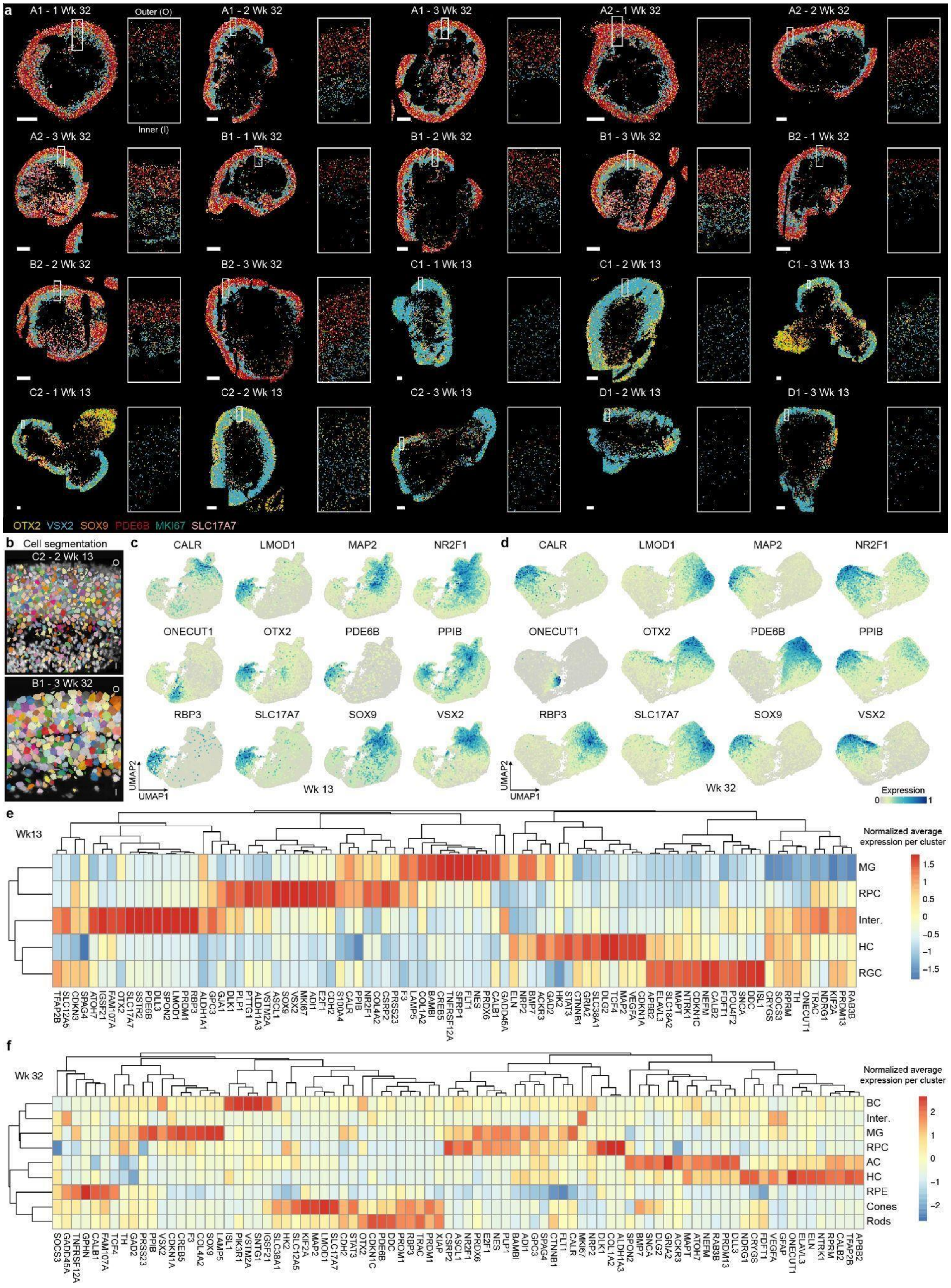
a
A1 - 1 Wk 32
Outer (O)
Inner (I)
A1 - 2 Wk 32
A1 - 3 Wk 32
A2 - 1 Wk 32
A2 - 2 Wk 32
A2 - 3 Wk 32
B1 - 1 Wk 32
B1 - 2 Wk 32
B1 - 3 Wk 32
B2 - 1 Wk 32
B2 - 2 Wk 32
B2 - 3 Wk 32
C1 - 1 Wk 13
C1 - 2 Wk 13
C1 - 3 Wk 13
C2 - 1 Wk 13
C2 - 2 Wk 13
C2 - 3 Wk 13
D1 - 2 Wk 13
D1 - 3 Wk 13
OTX2 VSX2 SOX9 PDE6B MKI67 SLC17A7
b
Cell segmentation
C2 - 2 Wk 13
B1 - 3 Wk 32
O
I
c
CALR LMOD1 MAP2 NR2F1
ONECUT1 OTX2 PDE6B PPIB
RBP3 SLC17A7 SOX9 VSX2
UMAP2
UMAP1
Wk 13
d
CALR LMOD1 MAP2 NR2F1
ONECUT1 OTX2 PDE6B PPIB
RBP3 SLC17A7 SOX9 VSX2
UMAP2
UMAP1
Wk 32
Expression
0 1
e
Wk13
Normalized average expression per cluster
MG
RPC
Inter.
HC
RGC
1.5
1
0.5
0
−0.5
−1
−1.5
TFAP2B SLC12A5 CDKN3 SPAG4 ATOH7 IGSF21 FAM107A OTX2 SLC17A7 SSTR2 PDE6B DLL3 SPON2 LMOD1 PRDM1 RBP3 ALDH1A1 GPC3 GJA1 DLK1 PLP1 PTTG1 VSTM2A ALDH1A3 ASCL1 SOX9 VSX2 MKI67 ADI1 E2F1 CDH2 S100A4 CALR PPIB NR2F1 COL4A2 CSRP2 PRSS23 F3 LAMP5 COL1A2 BAMBI CREB5 TNFRSF12A SFRP1 FLT1 NES PRDX6 CALB1 GADD45A ELN NRP2 BMP7 ACKR3 GAD2 HK2 STAT3 CTNNB1 SLC38A1 GRIA2 DLG2 TCF4 MAP2 VEGFA CDKN1A APBB2 ELAVL3 SLC18A2 MAPT NTRK1 CDKN1C NEFM CALB2 FDFT1 POU4F2 SNCA DDC ISL1 CRYGS SOCS3 RPRM TH ONECUT1 TRAC NDRG1 KIF2A PRDM13 RAB3B
f
Wk 32
Normalized average expression per cluster
BC
Inter.
MG
RPC
AC
HC
RPE
Cones
Rods
2
1
0
−1
−2
SOCS3 GADD45A TNFRSF12A GPHN CALB1 FAM107A TH TCF4 GAD2 PRSS23 PPIB VSX2 CDKN1A CREB5 F3 COL4A2 SOX9 LAMP5 ISL1 PIK3R1 VSTM2A SNTG1 IGSF21 SLC38A1 HK2 SLC12A5 KIF2A MAP2 LMOD1 SLC17A7 CDH2 STAT3 OTX2 CDKN1C PDE6B DDC PROM1 RBP3 TRAC PRDM1 XIAP CSRP2 ASCL1 NR2F1 PRDX6 E2F1 NES PLP1 BAMBI ADI1 GPC3 SPAG4 CTNNB1 FLT1 CALR MKI67 NRP2 DLK1 COL1A2 ALDH1A3 SPON2 BMP7 SNCA DLG2 GRIA2 ACKR3 MAPT ATOH7 NEFM RAB3B PRDM13 DLL3 NDRG1 CRYGS FDFT1 VEGFA GFAP ONECUT1 ELN NTRK1 ELAVL3 RPRM CALB2 TFAP2B APBB2

Supplemental Fig. 10: Multiplexed spatial transcript cartography in retinal organoids. a) All sections and organoids (week 32 and 13) profiled by multiplexed detection of RNA transcripts *in situ* (Molecular Cartography) are shown. An overlay of 6 transcripts (out of 100 probed) is shown. Scale bar = 100 µm, size of zoomed in windows 55.2x165.6 µm. b) Nuclei segmentation (Baysor) for a week 13 (top) and 32 (bottom) representative window with inner (I) and outer (O) orientation noted. c-d) Feature plots showing scaled gene expression for week 13 (c) and week 32 (d) organoid nuclei. e-f) Heatmaps showing z-scaled average expression of representative marker gene expression in week 13 (e) and week 32 (f) organoids for each major cell type.

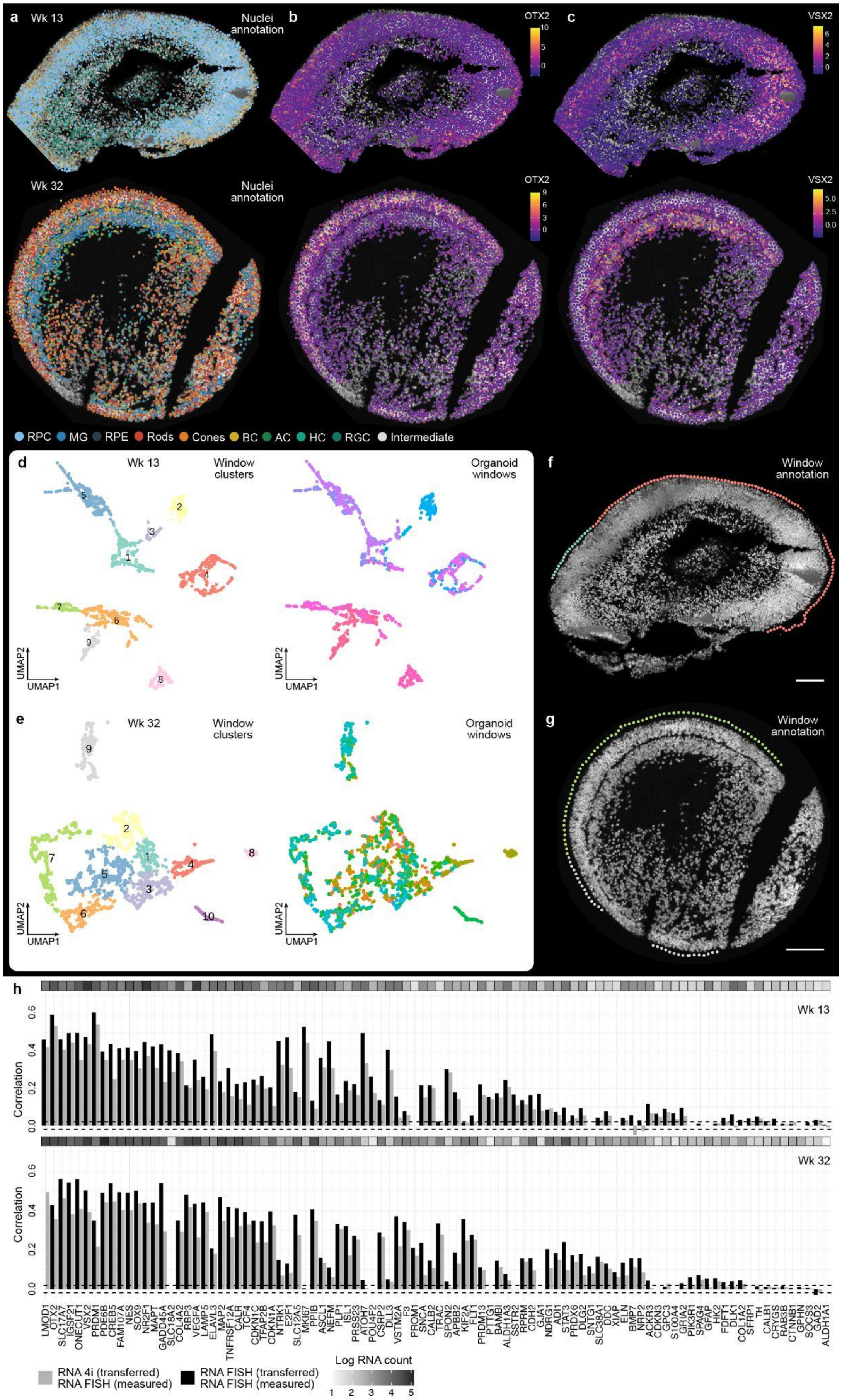

a
Wk 13
Nuclei annotation
b
OTX2
c
VSX2
Wk 32
RPC
MG
RPE
Rods
Cones
BC
AC
HC
RGC
Intermediate
d
Wk 13
Window clusters
Organoid windows
UMAP1
UMAP2
e
Wk 32
f
Window annotation
g
h
Correlation
LMOD1 OTX2 SLC17A7 IGSF21 ONECUT1 VSX2 PRDM1 PDE6B CREB5 FAM107A NES SOX9 NR2F1 MAPT GADD45A SLC18A2 COL4A2 RBP3 VEGFA LAMP5 ELAVL3 MAP2 TNFRSF12A CALR TCF4 CDKN1C TFAP2B CDKN1A NTRK1 E2F1 SLC12A5 MKI67 PPIB ASCL1 NEFM PLP1 ISL1 PRSS23 ATOH7 POU4F2 CSRP2 DLL3 VSTM2A F3 PROM1 SNCA CALB2 TRAC SPON2 APBB2 KIF2A FLT1 PRDM13 PTTG1 BAMBI ALDH1A3 SSTR2 RPRM CDH2 GJA1 NDRG1 ADI1 STAT3 PRDX6 DLG2 SNTG1 SLC38A1 DDC XIAP ELN BMP7 NRP2 ACKR3 CDKN3 GPC3 S100A4 GRIA2 PIK3R1 SPAG4 GFAP HK2 FDFT1 DLK1 COL1A2 SFRP1 TH CALB1 CRYGS RAB3B CTNNB1 GPHN SOCS3 GAD2 ALDH1A1
RNA 4i (transferred)
RNA FISH (measured)
RNA FISH (transferred)
RNA FISH (measured)
Log RNA count
1 2 3 4 5

Supplemental Fig. 11: Comparison between multimodal nuclei integrated with protein-transcript or transcript-transcript correlations. a-c) Nuclei annotation (a) and *OTX2* (b, left) and *VSX2* (c, right) transcript detection in a representative week 13 (top) and 32 (bottom) organoid section. d-e) UMAP of laminar windows from all week 13 (d) and 32 (e) organoid FISH data colored and numbered by cluster (left) or organoid section (right). f-g) Week 13 (f) and 32 (g) organoid sections with window annotation (colored dots) around the periphery colored by cluster. h) Barplot shows spatially constrained correlation between predicted and measured RNA for week 13 (top) and 32 (bottom) organoids (see Methods). Black represents correlation between predicted transcripts in integrated nuclei from the 4i experiment and *in situ* measured transcripts. Grey represents correlation between predicted transcripts in integrated nuclei from the FISH experiment and *in situ* measured transcripts. Top heatmaps show the averaged log transformed transcript count of each gene.

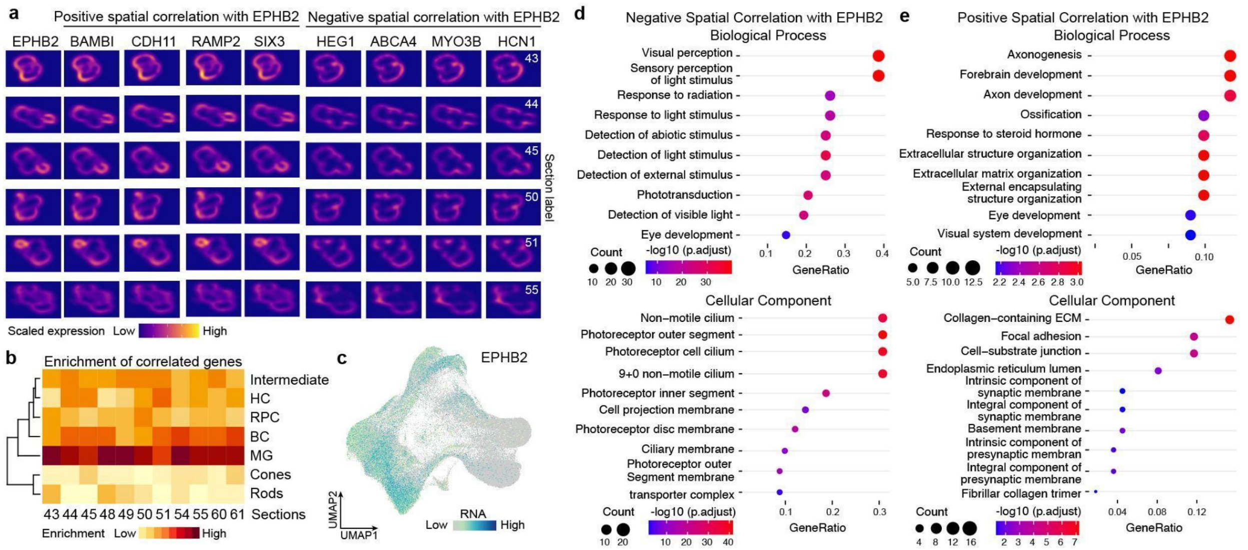

Supplemental Fig. 12: Identification of spatially-correlated transcriptome features associated with EPHB2 positive zones. a) Heatmaps from different sections of multiple 39 week organoids showing EPHB2 expression (left column) and genes that positively or negatively correlate with the EPHB2 spatial pattern. b) Heatmap showing spatial correlation of EPHB2 with each of the retinal organoid cell types across all week 39 sections. c) Feature plot showing expression of EPHB2 in the retinal organoid time course. d-e) Gene ontology Biological Process (top) or Cellular Component (bottom) enrichments (BH-corrected one-sided Fisher's exact test) for the top 115 genes that negatively (d) or positively (e) correlate with EPHB2 spatial domains.

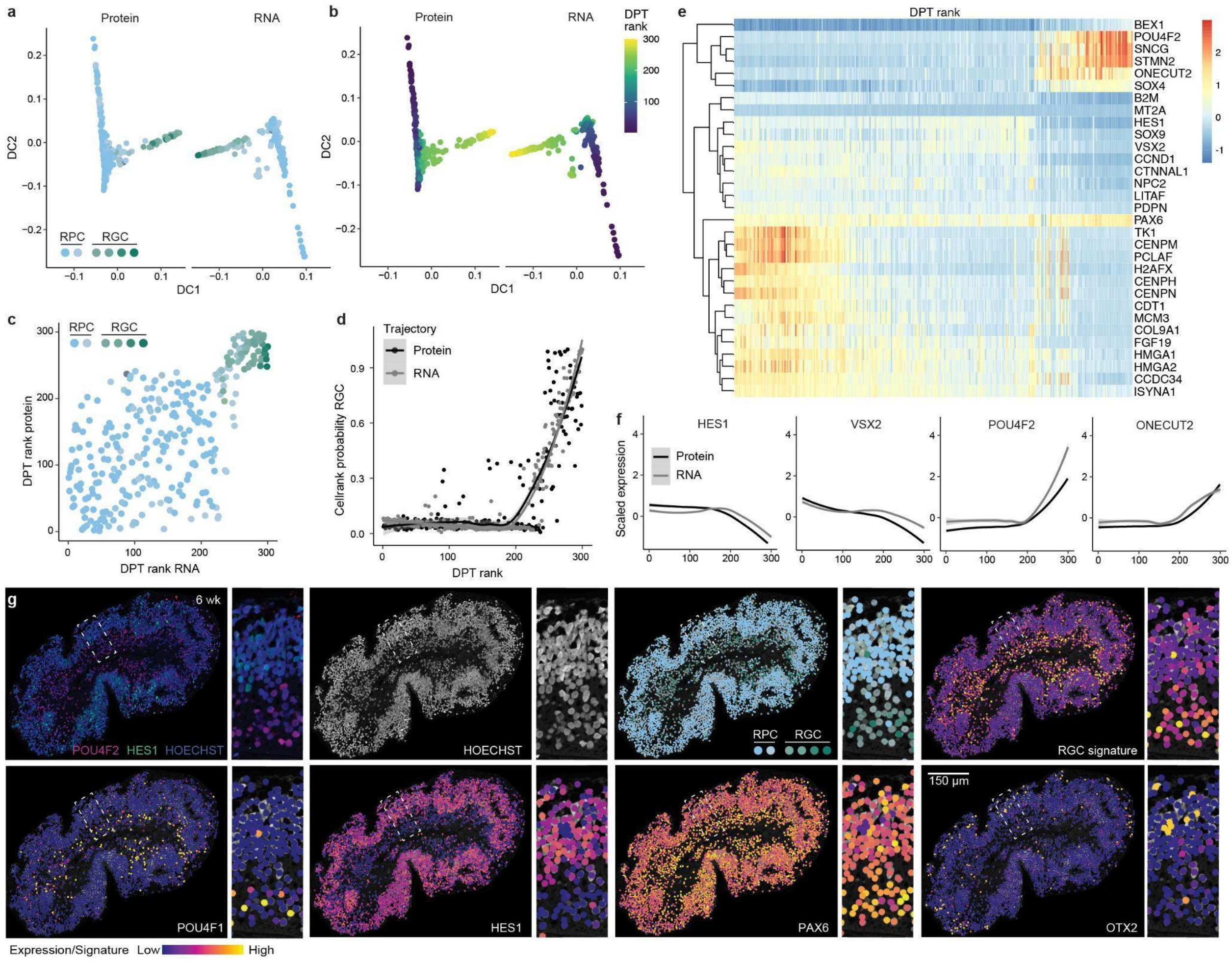

Supplemental Fig. 13: Analysis of retinal ganglion cell differentiation. a) Metacell projection in diffusion component space based on protein (left) or RNA (right) features. Metacells colored based on cell type (retinal progenitor cell, RPC, blue shades; retinal ganglion cell, RGC, green shades). b) Metacells colored based on pseudotime. c) Comparison of diffusion pseudotime (DPT) rank based on ordering from RNA (x axis) or protein (y axis) features. d) Quantification of RGC probability from the cellRank algorithm in each metacell shown across pseudotime. e) Heatmap shows normalized transcript abundance (z-score) profiles for pseudotemporally variable genes. f) Scaled expression of representative transcript expression based on protein (black) or RNA (grey) ordering. g) Organoid section (6 weeks) with a zoom to a representative window showing protein (POU4F2, HES1) and nuclei (HOECHST) features, RPC and RGC nuclei annotations, RGC signature per nuclei, and POU4F1/HES1/PAX6/OTX2 transcript expression per nuclei.

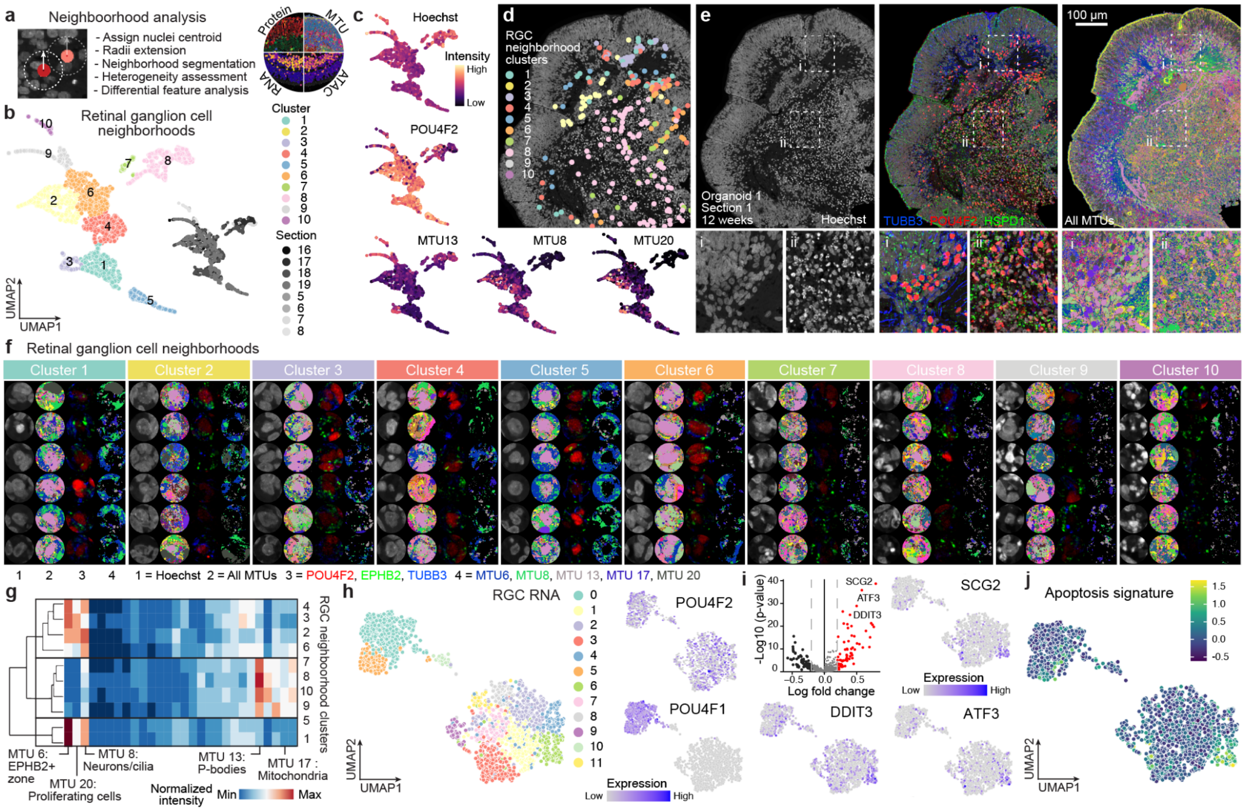

Supplemental Fig. 14: Analysis of retinal ganglion cell neighborhoods. a) Description of the neighborhood analysis algorithm. Cell neighborhoods were segmented through a 6.5 µm radial extension from each nuclei centroid, and then searched for heterogeneity among neighborhoods based on annotated features (e.g. MTUs, protein, RNA, chromatin access). b) UMAP embedding of each RGC neighborhood colored and numbered by cluster or by organoid section (inset, greyscale). Embedding is based on variation in the MTU space. c) RGC neighborhood UMAP colored by various cluster features. d) A representative 12 week organoid with RGCs nuclei colored by cluster. e) Nuclei stain (left), immunohistochemistry (middle), and MTUs (right) of a representative 12 week organoid across the time course. POU4F2 expression (red) marks retinal ganglion cells (RGCs). Nuclei located towards the interior of the organoid are stained more intensely due to condensation and often fragmented, indications of apoptosis (nuclei inset). f) Six random exemplary RGC neighborhoods per cluster with nuclei, MTUs and protein stains highlighted. g) Heatmap shows hierarchical clustering of RGC clusters based on MTU intensities, with selected MTUs shown and provisionally labeled. h) RGC heterogeneity was analyzed in the transcriptome space. UMAP embedding shows RGC transcriptomes colored by cluster (left), or by RGC marker expression (right). i) Differential gene expression (DE) analysis between clusters and gene ontology enrichment revealed a signature (genes colored in red) in cluster 6 that is highly enriched for apoptosis biological process terms. The volcano plot shows the result of DE analysis (using BH-corrected two-sided Wilcoxon test) between cluster 6 and other clusters, and feature plots show expression of top DE genes. j) RGC UMAP colored by the apoptotic signature (module score based on the expression of cluster 6 DE genes).

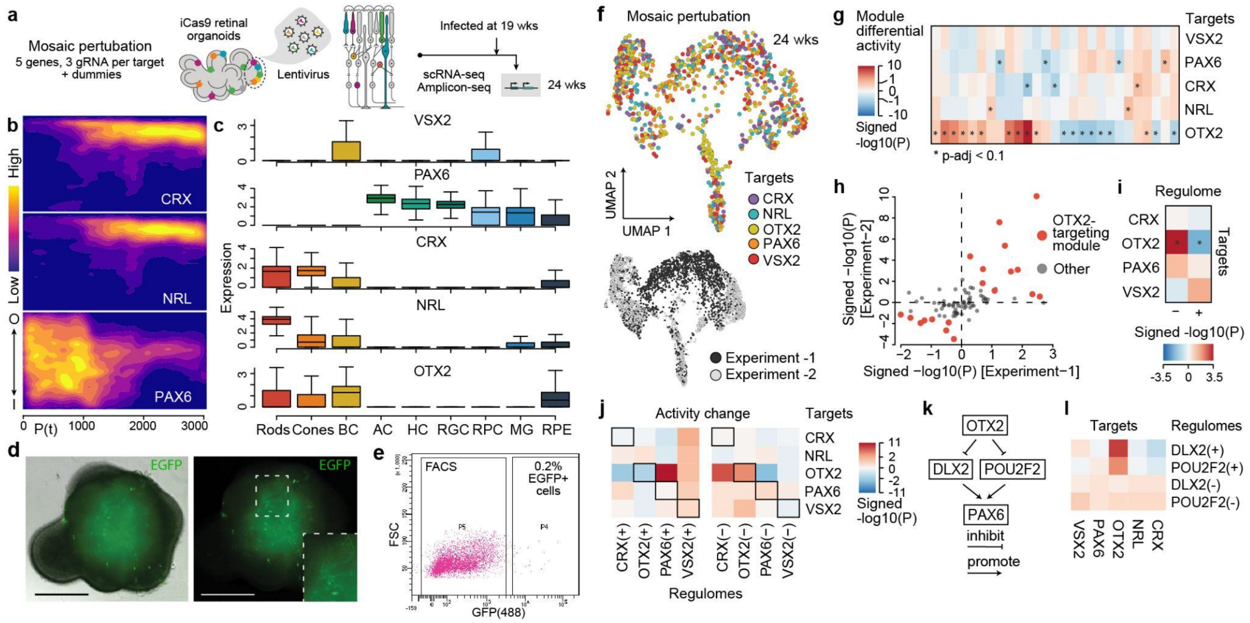

Supplemental Fig. 15: Analysis of the *in organoid* CROP-seq single-cell perturbation experiment in developing retinal organoids. a) Schematic of the mosaic perturbation experiment. b) Expression densities along the inner-outer and pseudotime axes of targeted TFs used in the CROP-seq experiment. c) Cell type expression of targeted TFs between 19-24 weeks of retinal organoid development. d) Representative retinal organoid infected with lentivirus containing gRNAs and EGFP reporter. Scale bar = 500 µm. e) Flow cytometry plot showing criteria for fluorescence activated cell sorting (FACS) in the perturbation experiment. f) UMAP embedding with cells colored by the detected gRNA (excluding cells without detected gRNA or with the dummy gRNA) (top) and by experiment (bottom). g) Differential module activity (using BH-corrected one-sided F-test, ANOVA) in TF-gRNA targeted cells relative to dummy control in the single cell time course, asterisks mark significantly differential activity. h) Scatter plot of module analysis correlation between the two CROP-seq experiments. i) Effects of gRNA targeted TFs on their predicted regulomes. j) Heatmap showing effects of each targeted TF in the CROP-seq experiment on the predicted regulomes of the other targeted TFs. k) GRN prediction of *PAX6* regulation by OTX2. l) Heatmap showing the effect of the CROP-seq targeted genes on the DLX2 and POU2F2 regulomes.
