# Supplementary material for: Multimodal spatiotemporal phenotyping of human retinal organoid development
Source: Nat Biotechnol. 2023 May 8;41(12):1765–75. doi: 10.1038/s41587-023-01747-2 (PMC10713453; doi:10.1038/s41587-023-01747-2)
Supplement: Supplementary file 2 — Reporting Summary [file 41587_2023_1747_MOESM2_ESM.pdf]

Corresponding author(s): Lucas Pelkmans
Barbara Treutlein
Jarret Grayson Camp

Last updated by author(s): 27.01.2023

Reporting Summary

Nature Research wishes to improve the reproducibility of the work that we publish. This form provides structure for consistency and transparency in reporting. For further information on Nature Research policies, see our Editorial Policies and the Editorial Policy Checklist.

Statistics

For all statistical analyses, confirm that the following items are present in the figure legend, table legend, main text, or Methods section.

n/a	Confirmed	
☐	☒	The exact sample size (*n*) for each experimental group/condition, given as a discrete number and unit of measurement
☐	☒	A statement on whether measurements were taken from distinct samples or whether the same sample was measured repeatedly
☐	☒	The statistical test(s) used AND whether they are one- or two-sided<br>*Only common tests should be described solely by name; describe more complex techniques in the Methods section.*
☐	☒	A description of all covariates tested
☐	☒	A description of any assumptions or corrections, such as tests of normality and adjustment for multiple comparisons
☐	☒	A full description of the statistical parameters including central tendency (e.g. means) or other basic estimates (e.g. regression coefficient) AND variation (e.g. standard deviation) or associated estimates of uncertainty (e.g. confidence intervals)
☐	☒	For null hypothesis testing, the test statistic (e.g. *F*, *t*, *r*) with confidence intervals, effect sizes, degrees of freedom and *P* value noted<br>*Give P values as exact values whenever suitable.*
☐	☒	For Bayesian analysis, information on the choice of priors and Markov chain Monte Carlo settings
☐	☒	For hierarchical and complex designs, identification of the appropriate level for tests and full reporting of outcomes
☐	☒	Estimates of effect sizes (e.g. Cohen's *d*, Pearson's *r*), indicating how they were calculated

Our web collection on statistics for biologists contains articles on many of the points above.

Software and code

Policy information about availability of computer code

Data collection	A detailed description of data collection is provided in the methods.<br><br>Genomics software tools used:<br>Cell Ranger (10x Genomics, v4.0.0), Cell Ranger ATAC (10x Genomics, v1.2.0), Cell Ranger ARC (10x Genomics, v2.0.0), MACS2 2.2.7.1, dropEst 0.8.6<br><br>Imaging acquisition software tools used: Zeiss Zen, NIS elements<br><br>Flow Cytometry software used: BD FACSAria™ III software
Data analysis	A detailed description of performed data analysis is provided in the methods.<br><br>Code used for image processing and data analysis of imaging and single-cell data sets is available on GitHub: https://github.com/quadbiolab/multimodal_retinal_organoids<br><br>Python packages used for image and single-cell data analysis:<br>scanpy 1.9.1, scVelo 0.2.5, CellRank 1.5.1, Cellpose 2.1.0, phenograph 1.5.7, fnnls 1.0.0, scikit-image 0.19.3, scikit-learn 0.24.1 , scipy ndimage 1.6.2, SimpleElastix 0.10.0<br><br>R packages used for single cell-analysis:<br>ggraph 2.1.0, igraph 1.3.5, presto 1.0.0, MASS 7.3-58.2, clusterProfiler 4.6.0, TSdist 3.7.1, DistatisR 1.1.1, destiny 3.12.0, Seurat 4.0.0, SeuratWrappers 0.3.1, Signac 1.9.0, Pando 1.0.3, Harmony 0.1.1, simspec 0.0.0.9000, FlowSOM 2.6.0, geneBasisR 0.0.0.9000

Other software used for image processing: Baysor 0.5.2, Fiji 2.9.0

For manuscripts utilizing custom algorithms or software that are central to the research but not yet described in published literature, software must be made available to editors and reviewers. We strongly encourage code deposition in a community repository (e.g. GitHub). See the Nature Research guidelines for submitting code & software for further information.

Data

Policy information about availability of data

All manuscripts must include a data availability statement. This statement should provide the following information, where applicable:

- Accession codes, unique identifiers, or web links for publicly available datasets
- A list of figures that have associated raw data
- A description of any restrictions on data availability

All the data can be visualized and explored via the EyeSee4is app (https://eyesee4is.ethz.ch/). Compressed images in JPEG format, an example 4i image dataset in the raw TIFF format, multiplexed smFISH spot data, processed datasets of integrated scRNA-ATAC, 4i nuclei and smFISH nuclei, as well as the 4i-scRNA-ATAC and smFISH-scRNA-ATAC integrated multimodal data are available via Zenodo (https://doi.org/10.5281/zenodo.7561908). The raw sequencing data is available via ArrayExpress under accession number E-MTAB-12622 (CROP-seq). The complete 4i image data of raw tiff files is too large to provide on public repositories. It is available upon request to the corresponding authors.

Field-specific reporting

Please select the one below that is the best fit for your research. If you are not sure, read the appropriate sections before making your selection.

☒ Life sciences ☐ Behavioural & social sciences ☐ Ecological, evolutionary & environmental sciences

For a reference copy of the document with all sections, see nature.com/documents/nr-reporting-summary-flat.pdf

Life sciences study design

All studies must disclose on these points even when the disclosure is negative.

Sample size

A detailed list of samples used for the 4i data is provided in Table S1. We aimed to include at least two biological replicates per time point and sample each replicate with 4 tissue slices. After quality control we kept at least two biological replicates and in all but two cases two cases two or more tissue sections per sample.
A detailed list of single-cell RNA-seq and single-cell ATAC is provided in Table S2. The analysis done in our project is as extensive as any previous study; based on Kanton et al. (Nature 2019) and Cowan, Renner (Cell, 2020) we believe that we sufficiently sample the heterogeneity with the number of cells sequenced.

Data exclusions

Tissue sections were excluded due to physical degradation or detachment from the glass slide during the 4i procedures. This resulted in incomplete stainings and preventer reasonable inclusion into the analysis design.
We excluded low quality cells using criteria as described in the Methods.

Replication

The 21 cycle 4i experiment presented was performed once. To verify replicability of the 4i results 4 kinds of controls were included into the study design.
1. The study includes a large number of replicates in one experiment. All Antibodies used were tested prior to the experiment and only included if they produced consistent stainings in several replicates. Antibodies that did not show the same staining pattern in the multi cycle experiment were excluded.
2. Elution controls were included to allow assessment of the elution efficacy of every antibody in several tissue sections matching the study samples.
3. The first cycle antibody stain was repeated after the completion of all cycles to assess the similarity of the stainings between the first and last cycle (overlay in Supplementary Fig. 2)
4. In half the samples the antibody order was permuted to allow assessment of effects of it in the stainings obtained.
We found all of these controls to verify the data quality. The assessment was based on visual examination.

The smFISH experiment was performed once including 4 randomly selected organoids at two timepoints.

For the time course data, we have 1-4 replicated samples for scRNA-seq (average 1.65), and 1-2 replicated samples for scATAC-seq (average 1.37) per time points. The scMultiome experiment has one sample per time point, two time points in total. The CROP-seq experiment have two replicated samples. In each sample, cells from 1-3 retinal organoids were pooled (see Supplementary Table 2).

Randomization

All organoids were selected at random from organoid batches.
For the 4i data every organoid was sampled by sections in equal ratios between the two groups of antibody order permutations. Sections were put onto the 96-well format coverslip in an order dictated by the microtome cutting procedure. The plate was split into the groups along the middle. Therefore the sections of one permutation are consistently ~10-20µm shifted within each sample. The samples were oriented randomly (organoids) or so as to allow retina cross sections (primary sample). We aimed to collect sections from the center planes of all organoids although we could not assess the centrality with certainty. Therefore in some organoids we have progressed further towards the center and in others beyond the center of the organoid as sectioning progressed. The consistency of our analysis confirms that there is no systematic bias between tissue sampling for the two groups.

Blinding

Blinding was not relevant to the study design as it did not include case and control samples. Investtigators were not blinded during data acquisition or analysis.

Reporting for specific materials, systems and methods

We require information from authors about some types of materials, experimental systems and methods used in many studies. Here, indicate whether each material, system or method listed is relevant to your study. If you are not sure if a list item applies to your research, read the appropriate section before selecting a response.

Materials & experimental systems

n/a	Involved in the study
☐	☒ Antibodies
☐	☒ Eukaryotic cell lines
☒	☐ Palaeontology and archaeology
☒	☐ Animals and other organisms
☐	☒ Human research participants
☒	☐ Clinical data
☒	☐ Dual use research of concern

Methods

n/a	Involved in the study
☒	☐ ChIP-seq
☐	☒ Flow cytometry
☒	☐ MRI-based neuroimaging

Antibodies

Antibodies used

A table listing all antibodies used and their dilution can be found in Table S1. The Antibody TUBB3 (Millipore, MAB1637) has the clone ID TU-20
Secondary antibodies Table_S1:
Host species Target species Channel wave length Company Order ID dilution
donkey goat 647 Abcam ab150135 1:500
goat chicken 647 Abcam ab150171 1:500
goat guineapig 647 Abcam ab150187 1:500
donkey mouse 568 Abcam ab175472 1:500
donkey rabbit 488 Abcam ab150073 1:500
donkey sheep 647 Abcam ab150179 1:500
goat rat 647 Abcam ab150159 1:500

Validation

We used Benchsci to select the most promising antibodies when available. If available we ordered knockout validated antibodies. All antibodies were tested before the multi cycle experiment by staining retinal organoid sections and comparing the results to manufacturere information. Antibodies were included if visual examination confirmed stainings similar to previously reported ones.

gene ID | Species | clonality | company | ID | report
DNM1 Ms monoclonal Thermo MA5-15285 https://app.benchsci.com/product/Thermo%20Fisher%20Scientific/MA5-15285/figures?product_type=antibody&ppage=1
SV2A Ms monoclonal Developmental Studies Hybridoma Bank NA https://app.benchsci.com/product/Developmental%20Studies%20Hybridoma%20Bank/DSHBSV2/info?product_type=antibody&ppage=1
NPC Ms monoclonal Abcam ab24609 https://app.benchsci.com/product/Abcam/AB24609/experimental_data?product_type=antibody&ppage=1
YAP1 Ms monoclonal Abcam ab56701 https://app.benchsci.com/product/Abcam/AB56701/info?product_type=antibody&ppage=1
TUBG1 Ms Monoclonal Sigma T5326 https://app.benchsci.com/product/Sigma-Aldrich/T5326/info?product_type=antibody&ppage=1
HSPD1 Ms monoclonal Abcam ab13532 https://app.benchsci.com/product/Abcam/AB13532/info?product_type=antibody&ppage=1
COL2A1 Ms monoclonal origene BM332 https://app.benchsci.com/product/Acris%20Antibodies/BM332/info?product_type=antibody&ppage=1
CCNE1 Ms monoclonal Abcam ab3927 https://app.benchsci.com/product/Abcam/AB3927/info?product_type=antibody&ppage=1
ACTB Ms monoclonal Abcam ab6276 https://app.benchsci.com/product/Abcam/AB6276/info?product_type=antibody&ppage=1
BSN Ms monoclonal Enzo SAP7F407 https://app.benchsci.com/product/Enzo%20Life%20Sciences/ADI-VAM-PS003-F/info?product_type=antibody&ppage=1
CTNNB1 Ms monoclonal BD Transduction Laboratories 610154 https://app.benchsci.com/product/BD%20Biosciences/610154/info?product_type=antibody&ppage=1
MAP2 Ms polyclonal Millipore AB5622 https://app.benchsci.com/product/Sigma-Aldrich/AB5622/info?product_type=antibody&ppage=1
PAX6 Ms monoclonal ATLAS AMAb91372 https://app.benchsci.com/product/American%20Research%20Products/AMAB91372/info?product_type=antibody&ppage=1
TUBB3 Ms monoclonal millipore MAB1637 https://app.benchsci.com/product/Sigma-Aldrich/MAB1637/info?product_type=antibody&ppage=1
GRIA2 Ms monoclonal Zymed 32-0300 https://app.benchsci.com/product/Thermo%20Fisher%20Scientific/32-0300/info?product_type=antibody&ppage=1
NEUROD1 Ms monoclonal Abcam ab60704 https://app.benchsci.com/product/Abcam/AB60704/info?product_type=antibody&ppage=1
TUBA4A Ms monoclonal Sigma T5168 https://app.benchsci.com/product/Sigma-Aldrich/T5168/info?product_type=antibody&ppage=1
RHO Ms monoclonal sigma R5403 https://app.benchsci.com/product/Sigma-Aldrich/R5403/info?product_type=antibody&ppage=1

RLBP1 Ms monoclonal Santz Cruz sc-59487 https://app.benchsci.com/product/Santa%20Cruz%20Biotechnology/SC-59487/info?product_type=antibody&ppage=1
CDH2 Sp polyclonal R&D Systems AF6426 https://app.benchsci.com/product/Novus%20Biologicals/AF6426/info?product_type=antibody&ppage=1
GLRA2 Gt polyclonal Santa Cruz Biotechnology sc-17279 https://app.benchsci.com/product/Santa%20Cruz%20Biotechnology/SC-17279/info?product_type=antibody&ppage=1
SLC32A1 Gp polyclonal Synaptic Systems 131004 https://app.benchsci.com/product/Synaptic%20Systems/131%20004/info?product_type=antibody&ppage=1
NRL Gt polyclonal R&D systems AF2945-SP https://app.benchsci.com/product/R%26D%20Systems/AF2945-SP/info?product_type=antibody&ppage=1
SLC17A7 Gp polyclonal Chemicon AB5905 https://app.benchsci.com/product/EMD%20Millipore/AB5905/info?product_type=antibody&ppage=1
Serotonin Gt polyclonal Abcam ab66047 https://app.benchsci.com/product/Abcam/AB66047/info?product_type=antibody&ppage=1
EPHB2 Gt polyclonal R&D Systems AF467 https://app.benchsci.com/product/Fisher%20Scientific/AF467/info?product_type=antibody&ppage=1
CALB1 Ck polyclonal Novus Biologicals NBP2-50028SS https://app.benchsci.com/product/Novus%20Biologicals/NBP2-50028SS/info?product_type=antibody&ppage=1
MAPT Gp polyclonal Synaptic Systems 314 004 https://sysy.com/product/314004
VSX2 Sp polyclonal exalpha X1180P https://app.benchsci.com/product/Exalpha%20Biologicals/X1180P/info?product_type=antibody&ppage=1
POU4F2 Gt polyclonal Santz Cruz sc-6026 https://app.benchsci.com/product/Santa%20Cruz%20Biotechnology/SC-6026/info?product_type=antibody&ppage=1
ONECUT2 Sp polyclonal R&D systems AF6294 https://app.benchsci.com/product/R%26D%20Systems/AF6294/info?product_type=antibody&ppage=1
MAPK1_MAPK3 Rb polyclonal Cell Signaling Technology 9101 https://app.benchsci.com/product/Cell%20Signaling%20Technology/9101/info?product_type=antibody&ppage=1
HES1 Rb monoclonal Cell signaling 11988 https://app.benchsci.com/product/Cell%20Signaling%20Technology/11988/info?product_type=antibody&ppage=1
PCNA Rb monoclonal Cell Signaling Technology 13110 https://app.benchsci.com/product/Cell%20Signaling%20Technology/13110/info?product_type=antibody&ppage=1
SOX9 Rb monoclonal Abcam ab185966 https://app.benchsci.com/product/Abcam/AB185966/info?product_type=antibody&ppage=1
GFAP Rb polyclonal abcam ab7260 https://app.benchsci.com/product/Abcam/AB7260/info?product_type=antibody&ppage=1
DDX6 Rb polyclonal Bethyl Laboratories A300-461A https://app.benchsci.com/product/Bethyl%20Laboratories/A300-461A/info?product_type=antibody&ppage=1
KCNJ10 Rb polyclonal Proteintech 12503-1-AP https://app.benchsci.com/product/Proteintech/12503-1-AP/info?product_type=antibody&ppage=1
CLUAP1 Rb polyclonal Proteintech 17470-1-AP https://app.benchsci.com/product/Proteintech/17470-1-AP/info?product_type=antibody&ppage=1
GPHN Rb polyclonal Chemicon (Millipore) AB5725 https://app.benchsci.com/product/Sigma-Aldrich/AB5725/info?product_type=antibody&ppage=1
GRIA4 Rb polyclonal Millipore AB1508 https://app.benchsci.com/product/EMD%20Millipore/AB1508/info?product_type=antibody&ppage=1
OPN1SW Rb polyclonal merck millipore AB5407 https://app.benchsci.com/product/EMD%20Millipore/AB5407/info?product_type=antibody&ppage=1
OPN1LW Rb polyclonal merck millipore AB5405 https://app.benchsci.com/product/EMD%20Millipore/AB5405/info?product_type=antibody&ppage=1
CALR Rb polyclonal Abcam ab2907 https://app.benchsci.com/product/Abcam/AB2907/info?product_type=antibody&ppage=1
AIPL1 Rb polyclonal ProteinTech 15108-1-AP https://app.benchsci.com/product/Proteintech/15108-1-AP/info?product_type=antibody&ppage=1
ATP1A1 Rb monoclonal abcam ab76020 https://app.benchsci.com/product/Abcam/AB76020/info?product_type=antibody&ppage=1
CTBP2 Rb polyclonal Synaptic Systems 192103 https://app.benchsci.com/product/Synaptic%20Systems/192%20103/info?product_type=antibody
MKI67 Rb monoclonal abcam ab16667 https://app.benchsci.com/product/Abcam/AB16667/info?product_type=antibody&ppage=1
PRKCA Rb monoclonal abcam ab32376 https://app.benchsci.com/product/Abcam/AB32376/info?product_type=antibody&ppage=1
IMPDH1 Rb polyclonal Proteintech 22092-1-AP https://app.benchsci.com/product/Proteintech/22092-1-AP/info?product_type=antibody&ppage=1
NES Rb polyclonal Sigma N5413 https://app.benchsci.com/product/Sigma-Aldrich/N5413/info?product_type=antibody&ppage=1
RCVRN Rb polyclonal merck millipore AB5585 https://app.benchsci.com/product/EMD%20Millipore/AB5585/info?product_type=antibody&ppage=1

Eukaryotic cell lines

Policy information about cell lines

Cell line source(s)	See methods p. 26<br>B7 (01F49i-N-B7 (Cowan, 2020))<br>IMR90 (iPS (IMR90)-4-DL-01, WiCell)<br>B7 iCas9 (01F49i-N-B7-iCas9 with integration of the construct pAAVS1-ieCas9)<br>409B2-iCas9 (He, 2021)
Authentication	See methods p.26
Mycoplasma contamination	Cell lines were tested for mycoplasma contamination on a regular basis using a PCR-based test and were founf to be negative for mycoplasma.

Commonly misidentified lines (See ICLAC register)	None.

Human research participants

Policy information about studies involving human research participants

Population characteristics	*Describe the covariate-relevant population characteristics of the human research participants (e.g. age, gender, genotypic information, past and current diagnosis and treatment categories). If you filled out the behavioural & social sciences study design questions and have nothing to add here, write "See above."*
Recruitment	*Describe how participants were recruited. Outline any potential self-selection bias or other biases that may be present and how these are likely to impact results.*
Ethics oversight	Ethikkommission Nordwest und Zentralschweiz (EKNZ)

Note that full information on the approval of the study protocol must also be provided in the manuscript.

Flow Cytometry

Plots

Confirm that:

- [x] The axis labels state the marker and fluorochrome used (e.g. CD4-FITC).
- [x] The axis scales are clearly visible. Include numbers along axes only for bottom left plot of group (a 'group' is an analysis of identical markers).
- [ ] All plots are contour plots with outliers or pseudocolor plots.
- [x] A numerical value for number of cells or percentage (with statistics) is provided.

Methodology

Sample preparation	Cell suspensions from infected organoids were obtained using a papain-based dissociation kit (Miltenyi Biotec, 130-092-628) as described in Methods (p. 31 and 39). 1ml of pre-warmed papain solution was added to the organoids and incubated for 10 minutes at 37 C. To facilitate dissociation, the mix was pipette-mixed every 5 minutes with a plO00. Enzyme mix A was then added and incubated for lOmin at 37C. At the end of the incubation, samples were pipette-mixed once more and successful tissue dissociation was confirmed via visual inspection. After incubation, cells were spun down 5min, 300g at 4 C. Cells were resuspended in 500µl of PBS+0.04%BSA and sequentially filtered through a 70µ m filter (pluriSelect Mini 101143-10070-50) and 40µm filter (pluriSelect Mini 43-10040-40) into a FACS tube for sorting.
Instrument	BD FACSAria Ill
Software	BD FACSAria software
Cell population abundance	The final sorted population was 0.2% of the total events.
Gating strategy	Three gates were used 1) FSC-A vs SSC-A was used to gate for the bulk population of cells, 2) FSC-W vs FSH-H was used to minimize doublet sorting, 3) GFP+ populations were gated and sorted as determined by comparing to not-infected wild type control populations.

- [x] Tick this box to confirm that a figure exemplifying the gating strategy is provided in the Supplementary Information.
